# TDF and TAF inhibit liver cancer cell migration, invasion via p7TP3

**DOI:** 10.1038/s41598-024-58807-z

**Published:** 2024-04-08

**Authors:** Jing Zhao, Li Zhou, Yang Zhang, Jun Cheng, Yilan Zeng, Xiuling Li

**Affiliations:** 1 Department of Gastoenterology, Henan Provincial People’s Hospital, People’s Hospital of Zhengzhou University, School of Clinical Medicine, Henan University, 7 Weiwu Rd, Zhengzhou City, 450003 Henan Province China; 2 Institiute of Infectious Diseases, Beijing Ditan Hospital, Capital Medical University/Beijing Key Laboratory of Emerging Infectious Diseases, Beijing, 100015 China; 3 Department of Infectious Disease, China-Japan Friendship Hospital, Beijing, 100029 China; 4 Chengdu Public Health Clinical Medical Center, Chengdu, 610061 China

**Keywords:** TDF/TAF, Liver cancers, p7TP3, Wnt/β-catenin, Drug discovery, Molecular biology, Gastroenterology

## Abstract

Tenofovir disoproxil fumarate (TDF) seems to prevent hepatocellular carcinoma (HCC) in patients with chronic hepatitis B virus (HBV). However, the mechanism is still little known. This study aimed to investigate the the roles and mechanisms of TDF, tenofovir alafenamide fumarate (TAF), and entecavir (ETV) on the malignant characteristics of liver cancer cells. Using the wound-healing assays, transwell assays, matrigel transwell assays, and cell counting kit-8 (CCK-8) assays, it was possible to identify that TDF/TAF, inhibited migration, invasion, and proliferation of HepG2 cells and Huh7 cells. To investigate the mechanisms, we performed TOP/FOP-Flash system, Western blot, and RT-qPCR assays of liver cancer cells cultured with TDF/TAF and found a lower activity of Wnt/β-catenin signaling pathway compared with control cells. Finally, Hepatitis C virus p7 trans-regulated protein 3 (p7TP3), a tumor suppressor in liver cancers, was significantly increased in HepG2 cells and Huh7 cells that treated with TDF/TAF. However, entecavir (ETV)-treated liver cancer cells showed no significant difference in the malignant characteristics of liver cancer cells, activity of Wnt/β-catenin signaling pathway, and expression of p7TP3, compared with the control groups. To conclude, TDF/TAF maybe novel promising therapeutic strategy for liver cancers, including HCC and hepatoblastoma, via Wnt/β-catenin signaling pathway, by up-regulating expression of the tumor suppressor, p7TP3.

## Introduction

Hepatocellular carcinoma (HCC), one of the most prevalent and deadliest cancers, has limited treatment options and poor patient prognosis^1^. The five-year survival rate of localized HCC is 31% and falls to 2% for metastatic HCC^2^. To prolong the length of life and improve its quality, the present article aims to contribute to the development of cancer research field and design of novel therapy approach.

Tenofovir disoproxil fumarate (TDF) and the newer Tenofovir alafenamide (TAF) are both pharmaceutical forms of tenofovir^3^. As nucleotide reverse transcriptase inhibitors, TDF and TAF are widely used for effective treatment of acquired human immunodeficiency virus and hepatitis B virus (HBV) infection in worldwide clinical settings^4^. Recent evidences suggest that TDF decrease the risk of HCC in patients with HBV^5,6^, reduce the HCC recurrence^7^, and lower the incidence of HCC in HIV/HBV-coinfected patients^8^. However, little is reported about the mechanisms of TDF in HCC. Besides, our previous researches have reported that TDF/TAF prevented progression and promoted reversion of liver fibrosis and pulmonary fibrosis^9,10^. Also, TDF/TAF inhibit inflammation in liver fibrosis^9^. In TDF-treated C57BL/6J mice, the differentially expressed genes (DEGs) were involved mainly in “immunity,” “inflammation,” and “metabolism” processes, comparing with control group^11^. Based on these studies, we suspect that TDF/TAF prevent progression of HCC, independently of its antiviral activity.

Previous research findings between the first-line agents for the treatment of chronic Hepatitis B (CHB) and HCC occurrences have been inconsistent and contradictory. Dates from South Korean national cohort^5,12^ and China national cohort^13^ demonstrate that TDF may confer a higher degree of protection from HCC, compared with entecavir (ETV). On the other hand, a 6-countries or regions cohort^14^ and another South Korean national cohort^15^ suggest that ETV and TDF show a comparable long-term risk of HCC. The roles of ETV, TDF, and TAF on clinical outcomes in CHB patients are controversial. At the same time, in this debate, there is a lack of voice for molecular biological mechanism researches. This paper attempts to show the discrepancies of ETV, TDF, and TAF on the malignant characteristics of liver cancer cells in vitro.

The p7TP3 gene, also known as TMEM50B, is located at 21q22.11, 447-bp long, and encodes a 158-residue protein^16,17^. P7TP3 may be a potential tumor suppressor gene in liver cancers. We have demonstrated that p7TP3 inhibit migration, invasion, adhesion, proliferation and cell cycle progression of liver cancer cells^17^. Besides, p7TP3 may be involved in malignant potential of thyroid nodules^18^. Moldrich et al. and Kong et al. identified p7TP3 significantly over-expressed in Down syndrome model, indicating that p7TP3 is important for correct brain development^19,20^. In this study, p7TP3 was confirmed as the target of TDF/TAF in liver cancers.

In HCC, Wnt/β-catenin signaling was frequently hyperactivated and promoted tumor growth and dissemination^21^. HepG2 cells, HSC39/40 cells, and hepatoblastomas have high β-catenin mediated transcriptional activity due to the presence of an interstitial deletion at the N-terminal region of β-catenin^22^. HepG2 is one of the specific cell lines concerning two forms of β-catenin, a wildtype protein and a truncated protein that is shorter than wild type^22^. While Huh-7 cells are known to be wild type for CTNNB1 (the β-catenin gene) and other genes of the Wnt pathway.

Based on these findings, we suspect that TDF/TAF may play a tumor suppressor role in liver cancer cells, including HCC cells and hepatoblastoma cells. Furthermore, we tried to explain why TDF/TAF may confer a higher degree of protection from liver cancer compared to ETV.

## Materials and methods

### Disclosure of ethical statements

This study did not involve animal experiments, human experiments, or human tissue samples. Ethics approval it is not relevant to this study.

### Materials

ETV, TDF, and TAF were friendship gifts from Anhui Biochem United Pharmaceutical Company and Chia Tai Tianqing (Nanjing, China).

### Cell culture and cell treatment

The HepG2 cell line was derived from a 15-year-old Caucasian human hepatocellular carcinoma. The Huh7 cell line was derived from a Japanese male with highly differentiated hepatocellular carcinoma. Human HCC cell line (Huh7 cells) and hepatoblastoma cell line (HepG2 cells) were provided by the Laboratory of Emerging Infectious Diseases at Beijing Ditan Hospital. The high-glucose Dulbecco's modified Eagle's medium (DMEM) (Gibco, Grand Island, NY, USA) were supplemented with 10% fetal bovine serum (Invitrogen Life Technologies), 100 U/ml penicillin, and 100 µg/ml streptomycin. Cells, being maintained in a humidified 5% CO_2_ and 37 °C incubator, were treated with TDF/TAF or the negative control, dimethyl sulfoxide (DMSO). DMSO was purchased from Sigma-Aldrich (MO, USA).

### The optimal concentrations of ETV, TDF, and TAF

According to the recommended clinical dose in healthy adults, the dosages of ETV, TDF, and TAF in mice models and cell cultures were identified (Table S1). To ensure clinical consistency and the safety of laboratory animals, we have standardized the administered doses. Based on the recommended clinical doses, the starting doses for first-in-mice trials were derived by the standardized process recommended by the Food and Drug Administration’s (FDA’s) guidance for industry^23^. The theoretical concentrations in vitro were calculated from the doses in vivo (5 μg/ml in vitro = 3 mg/kg in mice). The theoretical optimal concentrations of ETV, TDF and TAF in vitro were 0.508 μM, 130 μM, and 15.7 μM, respectively (Table S3). For the convenience of drug configuration, 0.50 μM, 125 μM, and 15 μM in vitro were selected in the concentration gradients. As expected in theory, the actual optimal concentrations in the experiment were 0.50 μM, 125 μM, and 15 μM.

### Wound healing assay

Cells were seeded into a 12-well plate that has been evenly labeled with 5 horizontal lines. Three horizontal line scratches perpendicular to horizontal lines were drawn by a 10 µl pipette tip, when the cells formed a tight cell monolayer. Washed the cell debris and added 1 ml of serum-free DMEM. Photos were recorded at 0, 24, 48, and 72 h, with the migration area being measured and the migration rate being calculated. The migration rate = (Initial wound area − Final wound area)/Initial wound area × 100%.

### Transwell migration assay and matrigel invasion assay

For transwell migration assay, cells in 200 µl of serum-free DMEM, were seeded into the upper transwell chamber (Corning, USA). The lower chamber contained 600 μl of DMEM containing 20% FBS. The recommended cell density was 1 × 10^5^. For matrigel invasion assay, 100 μl matrigel (1:5 dilution) was precoated chamber inserts and solidified in the incubator for 12 h. After being cultured for an additional 24 h, cells that did not pass through the transwell chamber were wiped off, while cells that migrated to surface of the lower membrane were fixed with ethanol and stained with crystal violet. Recorded photographs, and counted cells by Image J software.

### Proliferation assay

The cells were incubated with 1:10 diluted cell counting kit-8 (CCK-8) (Dojindo, Kumamoto, Japan). After incubation for 60 min in the dark at 37 °C, the optical density was measured at 450 nm.

### Western blot analysis

The protein concentration was determined strictly in accordance with the Pierce BCA Assay Kit (Thermo Scientific, USA) instructions. Samples were electrophoresed on 12% Bis–Tris Gel/MOPS (Invitrogen, NY, USA) for 2 h, and transferred to PVDF membranes (Immobilon, Millipore, USA) for 1.5 h. The membranes were incubated with 5% skimmed milk (2321000, BD, USA) for 1.5 h, incubated with the primary and secondary antibodies (Table S2). The antibodies for GAPDH^9^, Bcl-2^9^, Bax^9^, β-catenin^9^, and p7TP3^17^ has been used in previous experiments. The interested bands were analyzed by BiolD software (Vilber Lourmat, France). The blots were cut prior to hybridisation with antibodies. Therefore, original images of full-length blots cannot be provided. We have provided all original blots, with membrane edges visible, in supplementary materials.

### Quantitative reverse transcription polymerase chain reaction (RT-qPCR)

Total RNA was isolated from liver cancer cells using a total RNA Kit (Omega, USA). The single-stranded cDNA was synthesized from total RNA by cDNA Reverse Transcription Kit (Prime Script RT reagent Kit, TaKaRa, China), and subjected to RT-qPCR amplification (Applied Biosystems, USA). Primers were purchased from Su zhou Hong Xun (Table S3). The housekeeping gene β-actin was used to normalize and calculate relative expression ratios. The data were normalized to GAPDH for measuring the relative mRNA expression.

### TOP/FOP-flash system

TOP-flash plasmids and FOP-flash plasmids were transiently transfected into cells at a mass ratio of 50/1, with TDF, TAF or ETV being added to medium at the same time. After 24 h, cells were lysed and analyzed by dual-luciferase reporter assay kit (Promega, USA). Renilla luciferase vector plasmids in this system served as controls.

### Plasmids and short-interfering RNA (siRNA) oligonucleotides

The construction and amplification of pcDNA3.1/myc-His(−)-p7TP3 and pmirGLO-p7TP3 3′-UTR plasmids were performed as described in a previous study^17^. The short-interfering (siRNA) duplexes against human p7TP3 were purchased from GenePharma (343, Suzhou, China).

### Statistical analysis

All statistical analyses were performed using SPSS 17.0 software. Continuous variables from at least three independent experiments, are presented as the mean ± standard deviation, and were compared using Student’s *t* test. *P* < 0.05 was considered statistically significant.

## Results

### TDF and TAF inhibit malignant characteristics of liver cancer cells

**Figure 1:**
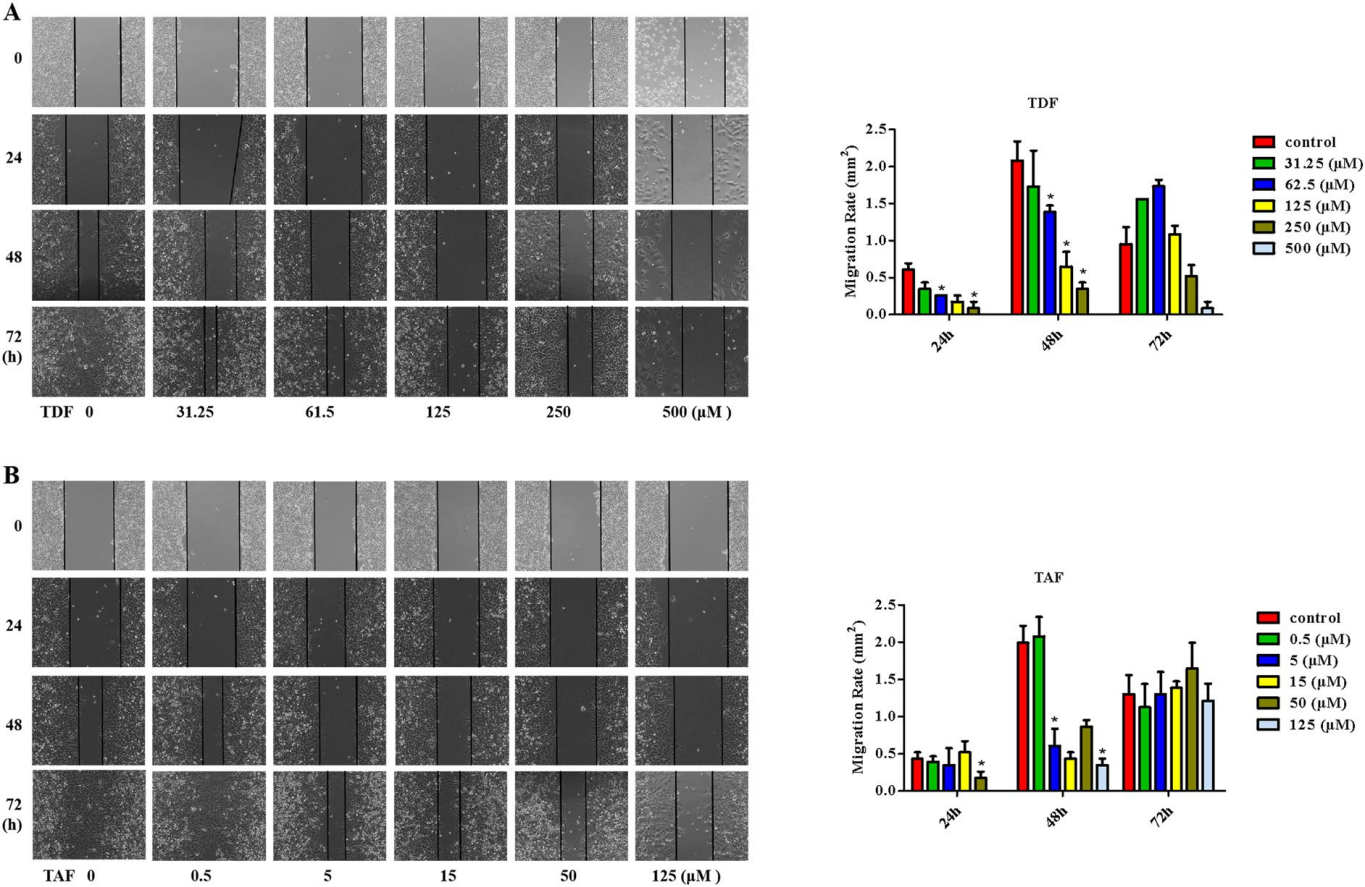
TDF/TAF inhibited liver cancer cell migration. HepG2 cells were scratched with straight lines using a 10 µl pipette tip, after cells were treated with different concentration of (**A**) TDF or (**B**) TAF for 24 h. Then the cell migration was detected, and the migration rate was calculated (*n* = 3), at 0, 24, 48 and 72 h (100 ×). The results shown are mean ± standard deviation. *P < 0.05.

**Figure 2:**
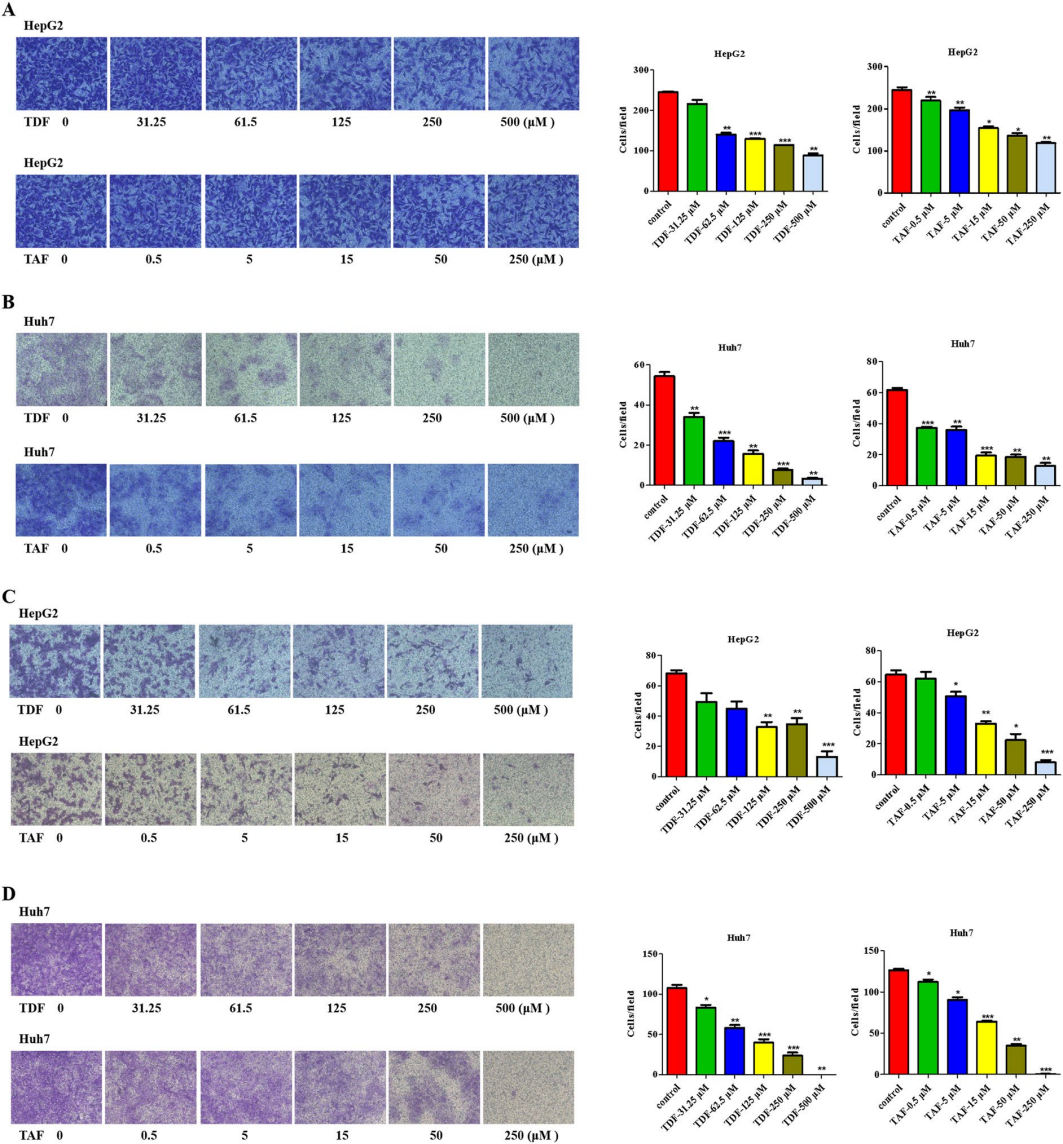
TDF/TAF inhibited liver cancer cell migration and invasion. Cells were treated with different concentration of TDF or TAF for 24 h. (**A**) HepG2 cells that treated with TDF or TAF were subjected to transwell assays for 24 h. A representative of three independent experiments was shown (200 ×). Then the transferred cells were counted by Image J soft, and migration rate was calculated (*n* = 3). (**B**) Migration rate of TDF/TAF-treated Huh7 cells were detected by the same method. (**C**) TDF/TAF-treated HepG2 cells were subjected to matrigel transwell assays for 24 h, (**D**) while TDF/TAF-treated Huh7 cells were for 48 h. Then the invasion rate was calculated (*n* = 3). The results shown are mean ± standard deviation. *P < 0.05, **P < 0.01, ***P < 0.001.

HepG2 cells treated with ETV, TDF, and TAF at different concentrations were used for wound healing assays (Fig. 1A,B) and transwell migration assays (Fig. 2A). The results indicated that the cell migration of TDF and TAF groups was greatly decreased compared with the control group, in a concentration-dependent manner at 24 and 48 h, whereas that of ETV showed no noticeable change (Fig. S1A,B). Similar results were also observed in Huh7 cells (Fig. 2B). These results suggest that the migratory capacities of liver cancer cells are significantly hindered by TDF and TAF.

We subsequently conducted matrigel invasion assays to detect the effects of TDF and TAF on invasive capacities. Results in TDF and TAF treated HepG2 cells showed notably lower invasive capacities, in concentration-dependent manners (Fig. 2C), compared with the control cells. The conclusion was also supported by assays in the Huh7 cell line (Fig. 2D). However, ETV-treated HepG2 cells showed no significant difference in the invasive ability, compared with the control cells (Fig. S1C). In Huh7 cells, only 0.5 μl of ETV prevented the invasive capacity (Fig. S1C). Together, these data reveal that TDF and TAF inhibit migration and invasion of liver cancer cells.

### TDF and TAF inhibit proliferation and facilitate apoptosis of liver cancer cells

**Figure 3:**
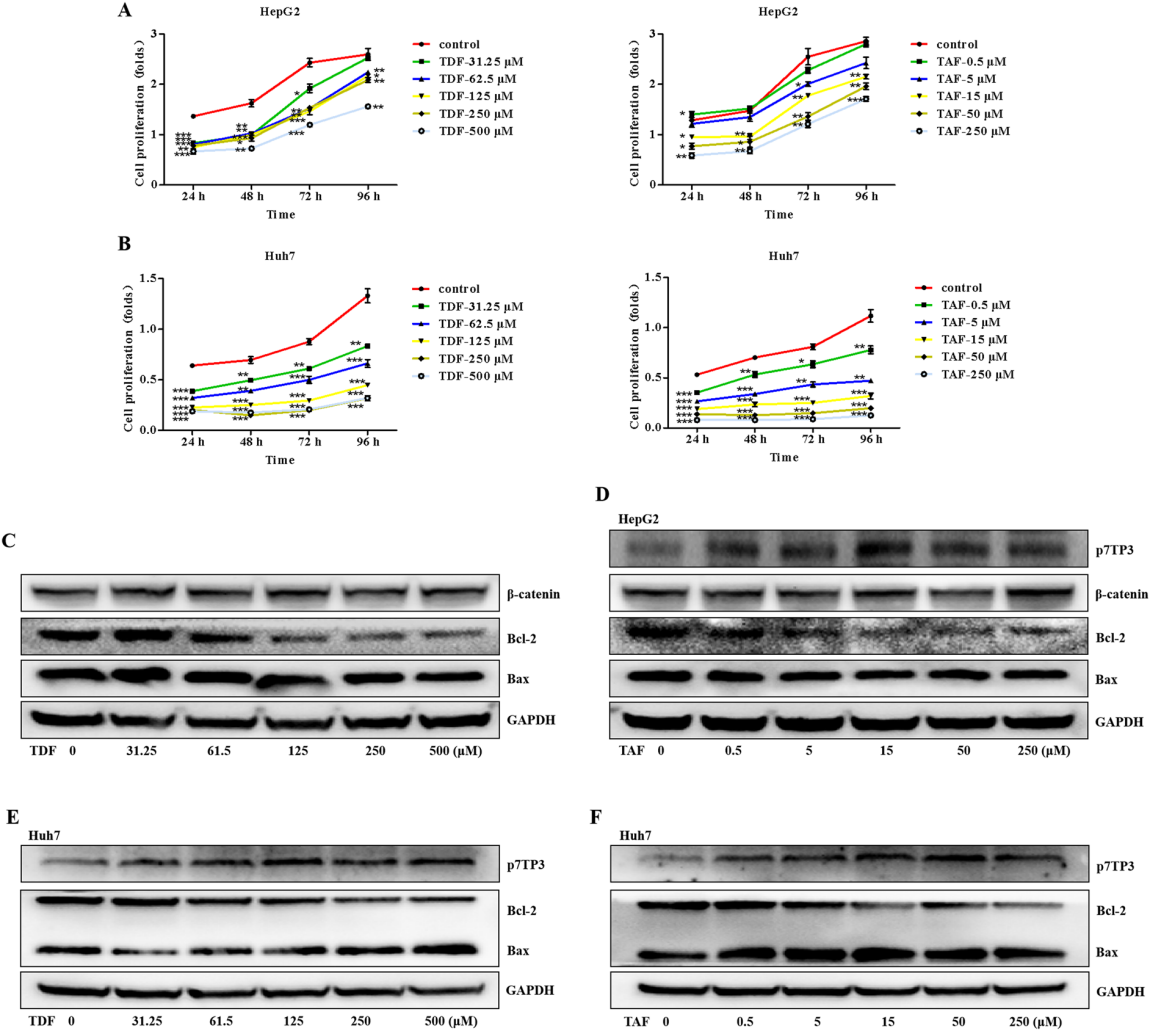
TDF/TAF inhibited liver cancer cell proliferation. Cells were incubated with fetal bovine serum-free medium, after stimulated with TDF/TAF for 24 h. Cells proliferation of (**A**) HepG2 cells and (**B**) Huh7 cells were evaluated by CCK-8 assays (*n* = 5). Total protein of in (**C**) TDF-HepG2 cells, (**D**) TAF-HepG2 cells, (**E**) TDF-Huh7 cells, (**F**) and TAF-Huh7 cells were extracted and immunoblotted after 48 h, with p7TP3, β-catenin protein, apoptosis-related genes Bcl-2 and Bax being analyzed. Original blots of (**C**, **D**) is presented in supporting Figs. S5–S10. Original blots of (**E**, **F**) is presented in supporting Figs. S11–S15. The results shown are mean ± standard deviation. *P < 0.05, **P < 0.01, ***P < 0.001.

To further investigate if TDF and TAF were involved in the proliferation ability of liver cancer cells, we first compared the cell viability by CCK-8 assays. HepG2 cells and Huh7 cells that were stimulated by different concentrations of ETV, TDF,or TAF, were subsequently cultured in FBS-free DMEM for 24, 48, 72, and 96 h. The results suggested that TDF and TAF markedly attenuated cell proliferation in concentration-dependent manners (Fig. 3A,B). Although ETV also inhibited cell proliferation, the inhibition seemed not closely related to the concentration gradient (Fig. S2A,B).

Bcl-2 is the anti-apoptosis gene, and its encoded protein inhibits apoptosis. Bax, as the pro-apoptotic gene, can be induced by p53 and has an inhibitory effect on tumors. The Bcl-2/Bax ratio plays a role in the balance of apoptosis. In this study, we analyzed the relationship between the Bcl-2/Bax ratio, using Western blot assays. Compared with the control group, the Bcl-2/Bax ratio was remarkably down-regulated by TDF and TAF, indicating that TDF and TAF promoted apoptosis of HepG2 cells in concentration-dependent manners (Fig. 3C,D). Similar results were also observed in Huh7 cells after TDF/TAF-treated (Figs. 3E,F). In contrast, ETV-treated HepG2 and Huh7 cells did not show significant changes, compared to the control group (Fig. S2C). Hence, our results suggest that TDF and TAF repress proliferation and promote apoptosis of liver cancer cells.

### TDF and TAF attenuate liver cancer cells progression by suppressing the Wnt/β-catenin signaling pathway

**Figure 4:**
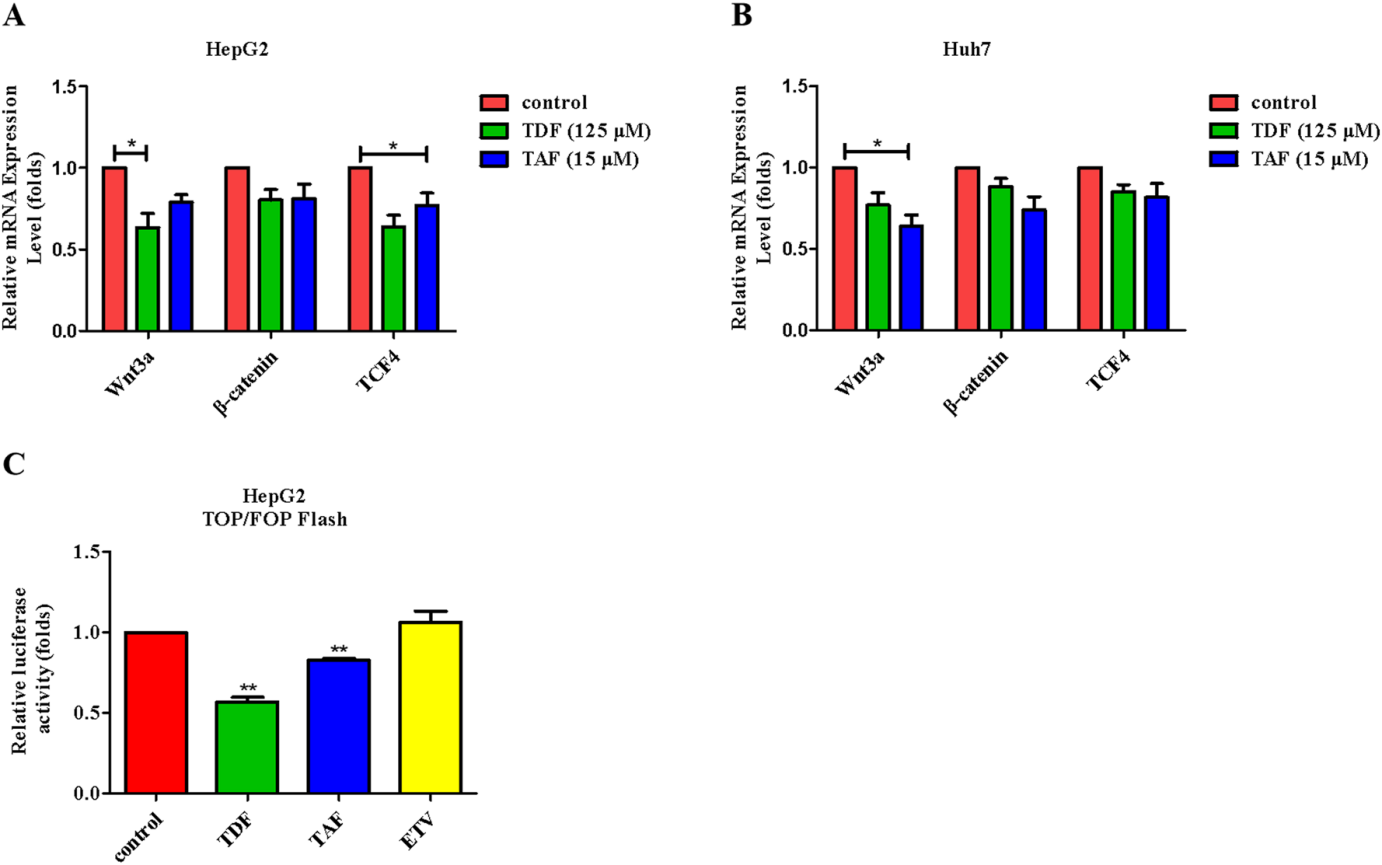
TDF/TAF inhibited Wnt/β-catenin signal pathway. Total protein and RNA were extracted, after HepG2 cells and Huh7 cells were treated with TDF/TAF for 48 h. (**A**) In HepG2 cells and (**B**) Huh7 cells, relative expression levels of Wnt/β-catenin signal pathway related molecules, Wnt3a, β-catenin, and TCF4 (*n* = 3). (**C**) HepG2 cells were transiently transfected with TOP-flash plasmids and FOP-flash plasmids (TOP-flash/TOP-flash = 50/1), together with TDF/TAF for 24 h. Activity of Wnt/β-catenin signal pathway was valued by dual-luciferase reporter assay (*n* = 3). The results are presented as mean ± standard deviation. *P < 0.05, **P < 0.01.

We found that TDF and TAF have no significant impact on β-catenin expression, both protein (Fig. 3C,D) and mRNA levels (Fig. 4A,B). Beta-catenin in the nucleus bind to the transcription factor TCF/LEF, initiating the transcription of downstream genes. We found that TDF/TAF significantly decreased expression of TCF4 in HepG2 cells and Huh7 cells (Fig. 4A,B). According to the optimal concentration above, in this experiment, the concentrations of TDF and TAF were 125 μM and 15 μM, respectively. The TOP/FOP FLASH system represented the transcriptional activity of genes triggered by Wnt/β-catenin signaling. The results confirmed that activity of Wnt/β-catenin signaling was simultaneously decreased when liver cancer cells were treated with TDF or TAF (Fig. 4C).

### TDF and TAF specifically promote the expression of p7TP3

**Figure 5:**
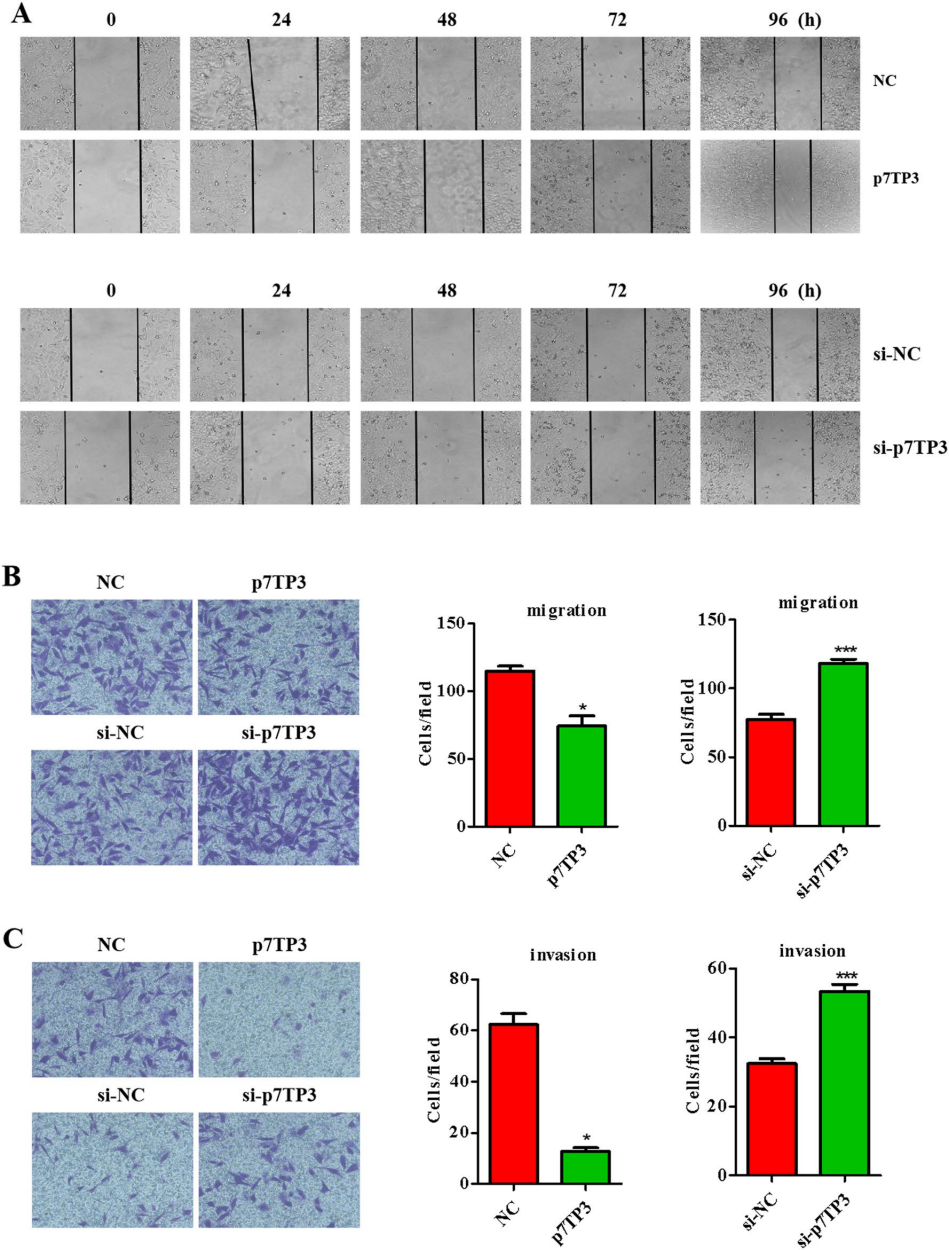
P7TP3 inhibited liver cancer cell migration and invasion. HepG2 cells were transiently transfected with pcDNA 3.1/myc-His(−)-P7TP3 plasmid, and siRNA-NC/siRNA-P7TP3, respectively. After 24 h, cells were subjected to (**A**) wound healing assays, (**B**) transwell migration assays and (**C**) transwell matrigel invasion assays (*n* = 3). The results shown are mean ± standard deviation. *P < 0.05, ***P < 0.001.

Wound healing assays (Fig. 5A) and transwell migration assays (Fig. 5B) showed that cell migration was significantly restrained by p7TP3, compared with control group, whereas that was promoted after p7TP3 silencing. In matrigel invasion assay (Fig. 5C), p7TP3 over-expression group showed a remarkable decrease in the invasive ability, while p7TP3 silencing group showed a significant increase.

**Figure 6:**
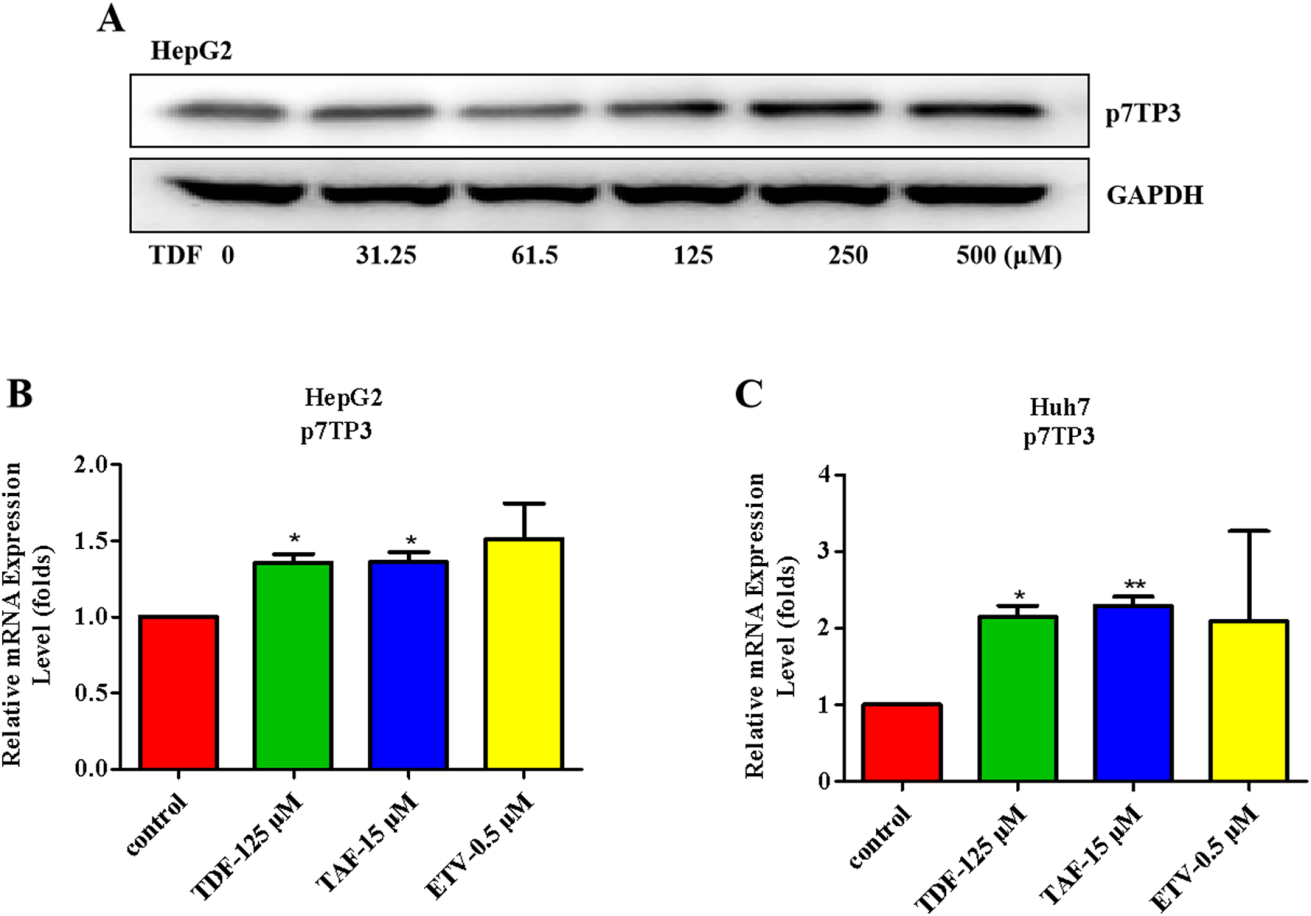
TDF/TAF up-regulated p7TP3 expression. Cells were treated with TDF/TAF for 48 h. (**A**) Western blotting analyse for p7TP3 protein in HepG2 cells. Original blots of (**A**) is presented in supporting Figs. S16 and 17. (**B**, **C**) Relative expression of p7TP3 in mRNA level (*n* = 3). The results shown are mean ± standard deviation. *P < 0.05, **P < 0.01.

Interestingly, we observed that TDF and TAF may up-regulate the expression of p7TP3 in liver cancer cells. To confirm whether p7TP3 expression is specifically up-regulated by TDF/TAF, we compared the expression profiles of p7TP3 in several nucleos(t) ide analogues (NAs) groups by western blotting assays, in HepG2 and Huh7 cells. The results revealed that p7TP3 expression was meaningfully up-regulated by both TDF and TAF, in a concentration-dependent manner (Figs. 3D–F and 6A), whereas p7TP3 expression was not altered by ETV (Figs. S3A,B). The real-time qPCR analysis (Fig. 6B,C) for p7TP3 gene were performed to determine the difference of p7TP3 expression in ETV, TDF, and TAF groups. These results demonstrate that p7TP3 maybe the target gene of TDF and TAF in liver cancers.

**Figure 7:**
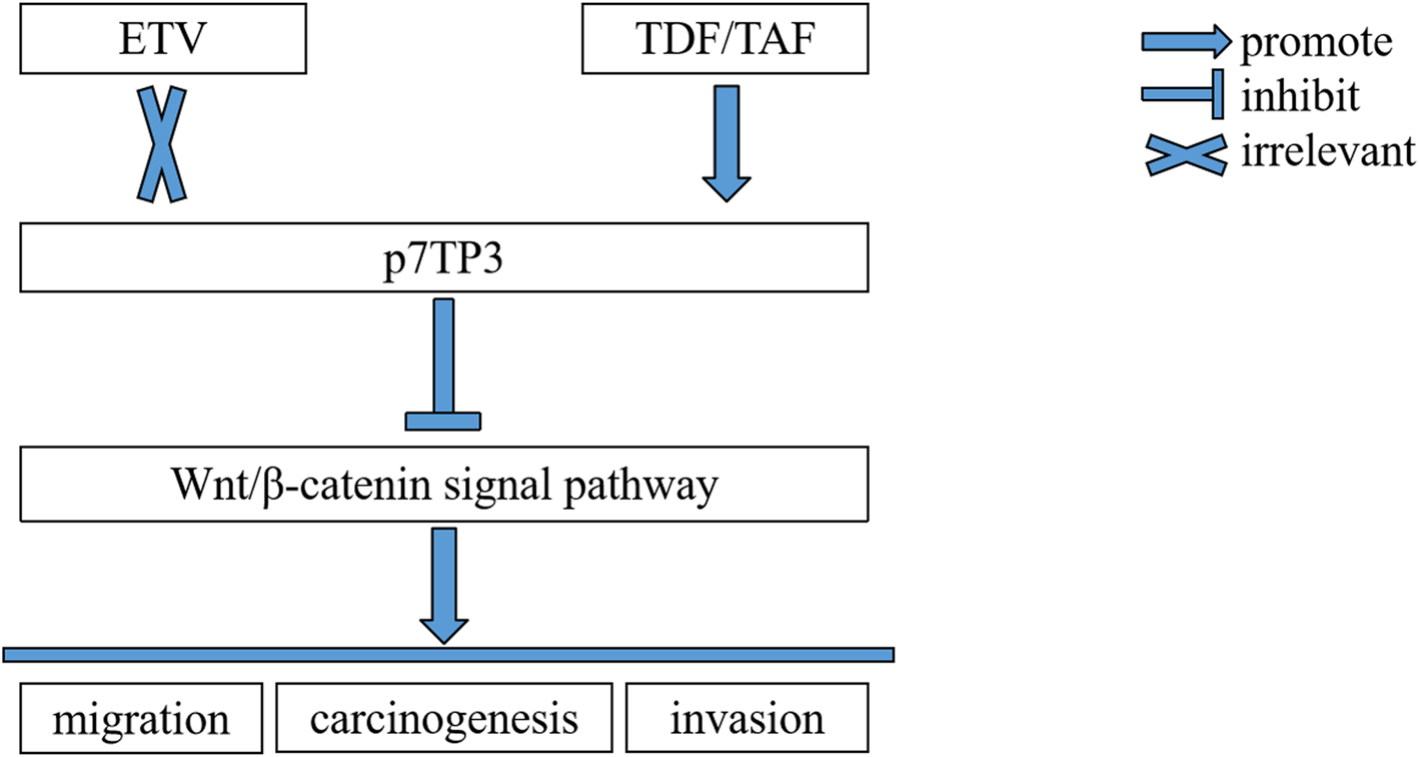
Schematic image.

Taken together, TDF and TAF inhibit migration, invasion, and proliferation in HepG2 and Huh7 cells. TDF and TAF suppress the Wnt/β-catenin signaling pathway activity. Finally, expression of p7TP3 is up-regulated by TDF and TAF (Fig. 7).

## Discussion

In this study, TDF and TAF were reported as novel anti-tumor drugs in vitro, independently of the antiviral activity. TDF and TAF are more beneficial to CHB clinical prognosis than ETV, probably because TDF and TAF inhibited migration, invasion, proliferation, and facilitate apoptosis of liver cancer cells by down-regulating Wnt/β-catenin signaling pathway, via targeting p7TP3. This study provide new insights on the role of TDF/TAF as antineoplastic agent in liver cancer cells.

Data suggested that TDF might decrease the risk of HCC in patients with HBV^5,6^. To explore the potential function of TDF/TAF in liver cancer cells, such as HepG2 cells and Huh7 cells, we conducted a series of assays in vitro*,* such as wound healing assays, transwell migration assays, matrigel invasion assays, and CCK-8 assays (Figs. 1, 2, 3). These studies showed that TDF/TAF inhibit migration, invasion, and proliferation of liver cancer cells, indicating that TDF/TAF inhibit malignant characteristics in liver cancer cells.

More interesting, TDF and TAF appear to inhibit liver cancers independently of the antiviral function. Crucially, cells in this study were hepatoma cell lines, HepG2 and Huh7 cell lines, which determined that all results had excluded hepatitis virus factors. On the other hand, TDF and TAF prevent progression and promote reversion in both liver fibrosis and pulmonary fibrosis in vivo^9,10^. These studies confirmed that TDF and TAF inhibit malignant characteristics of liver cancer cells, independently of antivirus. In addition, we observed that TDF and TAF suppressed the inflammatory response of hepatic fibrosis mice^9,10^, as well as mouse macrophage cell line, RAW264.7 cells (Fig. S4), suggesting that TDF and TAF may restrain progression of chronic liver disease by down-regulating inflammatory response.

TDF and TAF may inhibit malignant characteristics by suppressing inflammation. We examined the expression of the downstream molecules caspase-1 and interleukin-1β (IL-1β), in mouse macrophage cell line (Fig. S4), which represent the 3-containing NOD-like receptor pyrin domain (NLRP3) inflammatory body activity^21^. This study showed a significant increase in the activity of NLRP3 inflammasome in TDF/TAF-treated macrophage cell lines. The involvement of TDF/TAF in the inflammation needs further exploration. Persistent inflammation is known to promote and exacerbate malignancy, so we speculate that TDF/TAF suppressed liver cancers by inhibiting inflammation. To achieve effective therapy as well as prevention of liver cancers, this interesting conjecture needs further verification.

Moreover, TDF and TAF may inhibit malignant characteristics by suppressing the activaty of Wnt/β-catenin signaling pathway in vitro. We observed that TDF and TAF had no significant effects on β-catenin expression, protein, and mRNA levels (Figs. 3C,D and 4A,B), indicating that TDF and TAF may inhibit Wnt/β-catenin signaling activity by altering the stability of β-catenin rather than the total amount of β-catenin. Next, we performed TOP/FOP flash plasmid system to detect the transcription factor TCF/LEF that binds to nuclear β-catenin, which represents activation of the Wnt/β-catenin signaling pathway. Here, TOP/FOP-Flash reporter assay showed that TDF/TAF significantly decreased Wnt/β-catenin signaling activity (Fig. 4C).

By the suppression of subtractive hybridization (SSH) and yeast-two hybrid system, 127 new genes were screened, cloned, and registered at GenBank by us^24^. These new genes have been demonstrated to be closely related to liver diseases such as HCC^24^. Previous studies have shown that p7TP3 was a new tumor suppressor in HCC^17^. Here, we first reported that P7TP3 maybe the target gene of TDF and TAF in liver cancers, including HCC and hepatoblastoma.

This study was novel in identifying that p7TP3 expression was up-regulated by TDF and TAF in liver cancer cells. The optimal concentrations for TDF and TAF to up-regulate the expression of p7TP3 in protein level were 125 μM and 15 μM, respectively (Figs. 3D–F and 6A). Corresponding concentrations of TDF and TAF also significantly up-regulated the expression of p7TP3 in mRNA level (Fig. 6B,C). Interestingly, using western blot assays, we observed that p7TP3 expression was not regulated by ETV at the protein levels (Fig. S3). The discrepancy in the inhibition of HCC by ETV, TDF, and TAF may depend on differences in the p7TP3 expression. However, the specific mechanisms remain to be studied. However, in the current study, we did not provide direct evidence that if TDF/TAF inhibit liver cancers in the p7TP3 silencing model. We speculate that unraveling the puzzle is helpful for the postvention of chronic liver diseases and the treatment of liver cancers.

A controversial question is whether clinical outcomes differ in patients treated with ETV, TDF, and TAF regimens. In our study, we confirmed that TDF and TAF are more advantageous than ETV in constraining liver cancer cells migration, invasion, and proliferation, providing evidence that TDF and TAF provide benefits over ETV in liver cancer prevention. Unfortunately, our study lacked further validation in animal experiments. This interesting conjecture needs further verification.

In addition to the discussion above, this study has other limitations. First, not all experiments were performed simultaneously in both HepG2 and Huh7 cells. Human liver cancer cell lines are diverse and are used for varied experimental purposes. HepG2 cell line is derived from hepatoblastoma, while Huh7 cell line is derived from highly differentiated hepatocellular carcinoma. These experiments were conducted in two liver cancer cell lines simultaneously to confirm the data. Unfortunately, the cell adhesion ability of Huh7 cells remained weak, as it was suspended in cell culture dish after straight scratches were drawn with a 10 µl sterile pipette tip. So, wound healing assays are performed in HepG2 cells only (Fig. 1). The dual-luciferase reporter assays in Fig. 4C. This study was performed in HepG2 cells, but not Huh7 cells. Firstly, the Huh7 cells were sensitive to the external environment, so that the cells died after co-transfection of various reagents and plasmids of luciferase. In addition, the binding of the transcription factor to the promoter did not change because of changes in cell lines. Therefore, only HepG2 cells be used for dual-luciferase reporter assay.

The study has other limitations. First, does TDF/TAF inhibit liver cancers primarily by suppressing the inflammatory response? Secondly, what is the optimal dose and side effect of TDF/TAF as clinical anti-tumor drugs in vivo? Thirdly, how do TDF and TAF regulate p7TP3 expression? Fourthly, does TDF/TAF still inhibit liver cancers after potential target gene p7TP3 silencing?

In summary, we showed that TDF and TAF were superior to ETV in vitro as anti-tumor drugs. Assays demonstrated the significance of TDF and TAF in inhibiting liver cancer cells migration and invasion by targeting p7TP3. These encouraging results may offer a novel therapeutic strategy for liver cancers in the future.

### Supplementary Information

Supplementary Information.

## Data Availability

The datasets used and analysed during the current study available from the corresponding author on reasonable request.
