# Supplementary material for: TDF and TAF inhibit liver cancer cell migration, invasion via p7TP3
Source: Sci Rep. 2024 Apr 8;14:8161. doi: 10.1038/s41598-024-58807-z (PMC11001947; doi:10.1038/s41598-024-58807-z)
Supplement: Supplementary file 1 — Supplementary Information. [file 41598_2024_58807_MOESM1_ESM.pdf]

Supporting Figure Legends

Supporting Figure 1. ETV did not regulate liver cancer cell migration and invasion

(A) ETV-treated HepG2 cells were subjected to wound healing assays, with the migration rate being measured (*n* = 3). In HepG2 cells and Huh7 cells, migration rate and invasion rate were detected in ETV-treated, using (B) transwell migration assays and (C) transwell matrigel invasion assays (*n* = 3). The results shown are mean ± standard deviation. *P < 0.05.

Supporting Figure 2. ETV inhibited liver cancer cell proliferation

The effect of ETV on cells proliferation of were evaluated by CCK-8 assays in (A) HepG2 cells and (B) Huh7 cells (*n* = 5). Expression of Bcl-2 and Bax in protein level were detected by Western blotting (C). Original blots of Figure S2C is presented in supporting Figures 18-24. The results shown are mean ± standard deviation. *P < 0.05, **P < 0.01, ***P < 0.001.

Supporting Figure 3. ETV did not regulate p7TP3 expression

HepG2 cells and Huh7 cells were treated with different concentration of ETV for 48 h. (A) Western blotting for p7TP3 protein in HepG2 cells. (B) Western blotting for

p7TP3 protein in Huh7 cells. Original blots of Figure S3A is presented in supporting Figures 25-28.

Supporting Figure 4. TDF and TAF inhibit expression of caspase-1 and IL-1β

The mouse macrophage cell line, RAW264.7 cells, were used in this study. RAW264.7 cells were stimulated by 1 μg/mL of lipopolysaccharide (LPS) 12 h, prior to TDF/TAF treatment 24 h. Western blotting for caspase-1 and IL-1β protein in TDF group (A) and TAF group (B) . Original blots of Figure S4 is presented in supporting Figures 29-30.

Supporting Figure 5. Original blots of Bcl-2 in Figure 3C

Supporting Figure 6. Original blots of Bcl-2 in Figure 3D

Supporting Figure 7. Original blots of Bax in Figures 3C and D

Supporting Figure 8. Original blots of GAPDH in Figures 3C and D

Supporting Figure 9. Original blots of β-catenin in Figures 3C and D

Supporting Figure 10. Original blots of p7TP3 in Figures 3D

Supporting Figure 11. Original blots of Bcl-2 and Bax in Figures 3E

Supporting Figure 12. Original blots of Bcl-2 and Bax in Figures 3F

Supporting Figure 13. Original blots of GAPDF in Figures 3E and F

Supporting Figure 14. Original blots of p7TP3 in Figures 3E

Supporting Figure 15. Original blots of p7TP3 in Figures 3F

Supporting Figure 16. Original blots of p7TP3 in Figure 6A

Supporting Figure 17. Original blots of GAPDH in Figure 6A

Supporting Figure 18. Original blots of HepG2-Bax in Figure S2C

Supporting Figure 19. Original blots of HepG2-Bcl2 in Figure S2C

Supporting Figure 20. Original blots of HepG2-GAPDH in Figure S2C

Supporting Figure 21. Original blots of Huh7-Bax in Figure S2C

Supporting Figure 22. Original blots of Huh7-Bcl2 in Figure S2C

Supporting Figure 23. Original blots of Huh7-GAPDH for Bax in Figure S2C

Supporting Figure 24. Original blots of Huh7-GAPDH for Bcl2 in Figure S2C

Supporting Figure 25. Original blots of HepG2-GAPDH in Figure S3A

Supporting Figure 26. Original blots of HepG2-p7TP3 in Figure S3A

Supporting Figure 27. Original blots of Huh7-GAPDH in Figure S3B

Supporting Figure 28. Original blots of Huh7-p7TP3 in Figure S3B

Supporting Figure 29. Original blots of caspase1 and GAPDH in Figure S4

Supporting Figure 30. Original blots of IL1 in Figure S4

Supporting Tables

Supporting Table 1 The optimal concentrations of ETV, TDF, and TAF

Drugs \ Dosage	Human (mg/d)	Mice (mg/kg/d)	*In vitro* (μM)
ETV	0.5	0.09	0.508
TDF	300	50	130
TAF	25	4.5	15.7

Supporting Table 2 The primary antibodies used in the study

Antibodies name	Dilution ratio	Company name	Product code
anti-p7TP3	1:1000	Thermo Fisher	AA3B06N
anti-GAPDH	1:1000	CST	5174
anti-bcl-2	1:1000	CST	15071
anti-bax	1:1000	CST	5023
anti-β-catenin	1:1000	Abcam	ab32572
anti-caspase-1	1:1000	Abcam	ab1872
anti-IL-1β	1:1000	Abcam	ab9722

Supporting Table 3 Optimized primers used in the study

Genes	Sense (5′— 3′)	Antisense(5′— 3′)
P7TP3	atgcagctgtggtgtatcct	actcgagcacctgttcttcc
β-catenin	gctttcagttgagctgacca	caagtccaagatcagcagtctc
TCF-4	tctctgcaagagacttccatcc	agacaggtctgttgggagtg
Wnt3a	acttttgtgagcccaaccca	ttctccgtcctcgtgttgtg
β-actin	cactcttccagccttccttcc	cgtacaggtctttgcggatgtc

Supporting Figures

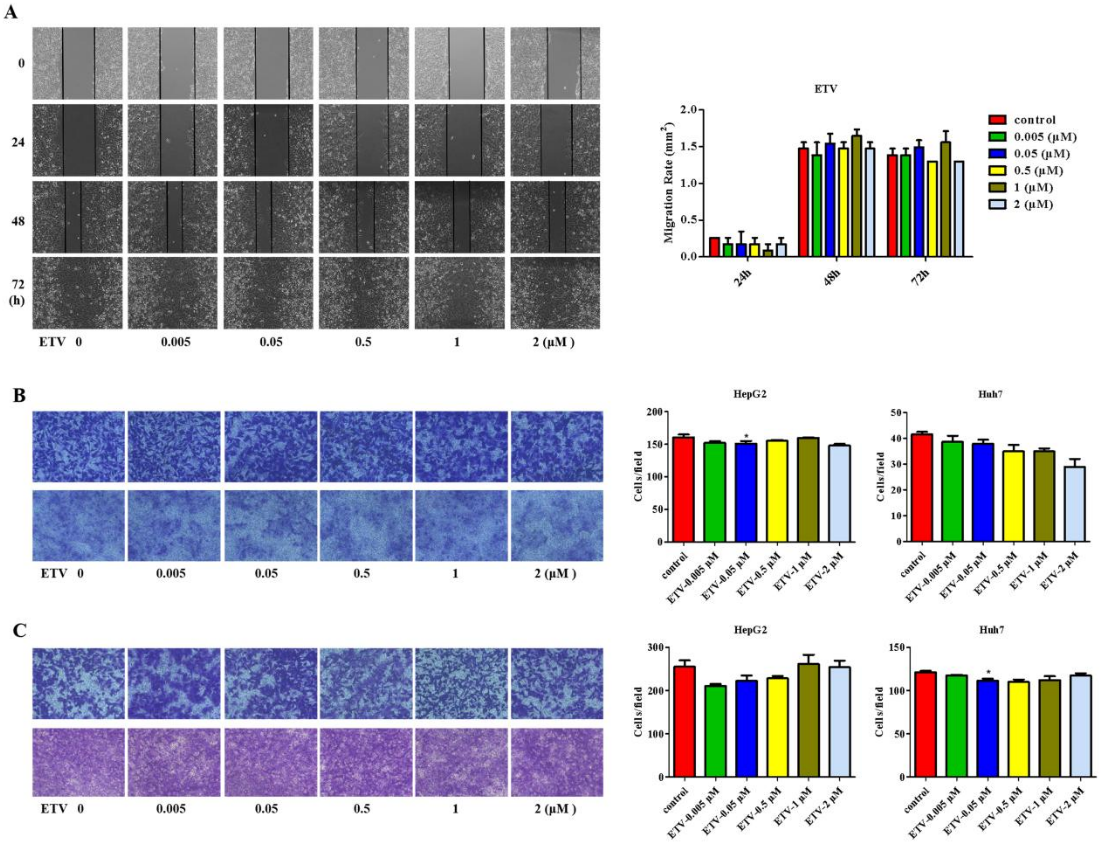

Supporting figure 1. ETV did not regulate liver cancer cell migration and invasion

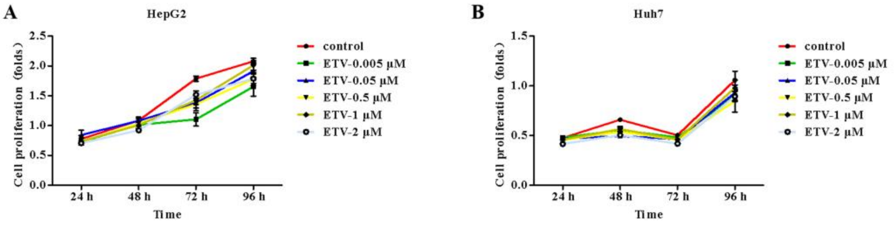

	0.005-μM	0.05-μM	0.5-μM	1-μM	2-μM
24 h					
48 h				**	
72 h		*	**	**	***
96 h			*		

	0.005-μM	0.05-μM	0.5-μM	1-μM	2-μM
24 h					*
48 h	**	***			***
72 h				*	**
96 h			*		*

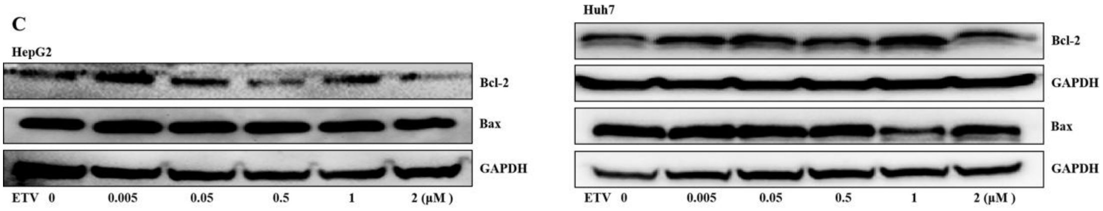

Supporting figure 2. ETV inhibited liver cancer cell proliferation

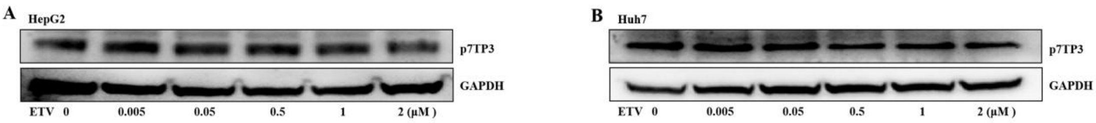

Supporting figure 3. ETV did not regulate p7TP3 expression

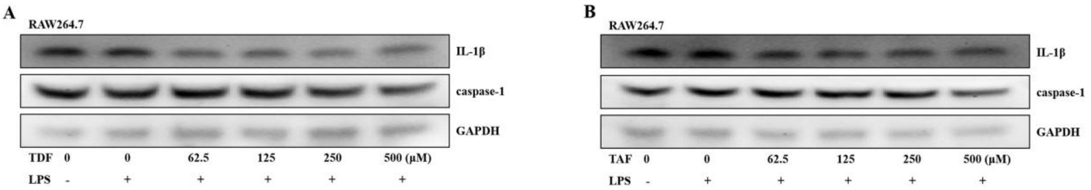

Supporting figure 4. TDF and TAF inhibit caspase-1 and IL-1β expression

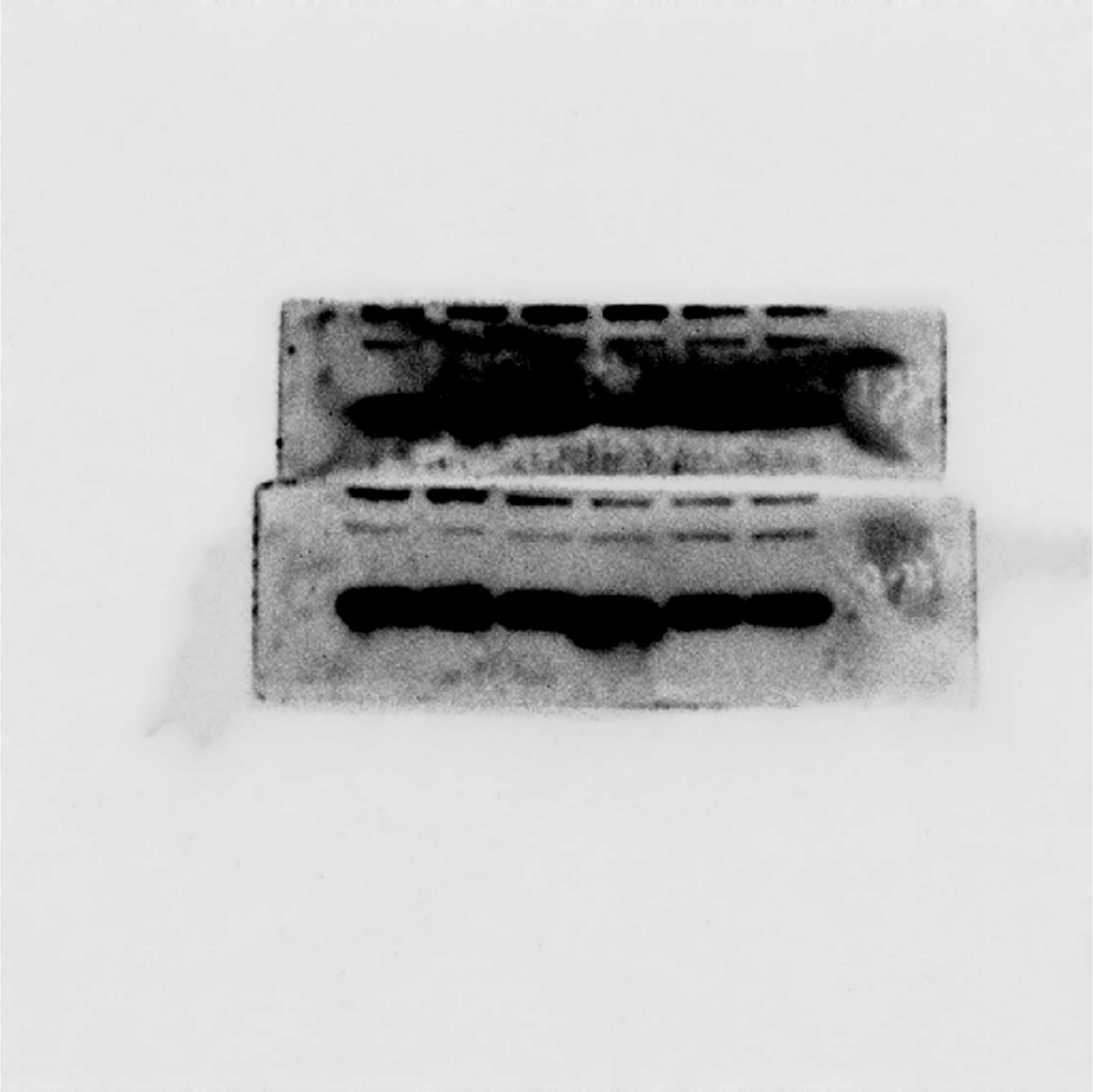

Supporting Figure 5. Original blots of Bcl-2 in Figure 3C

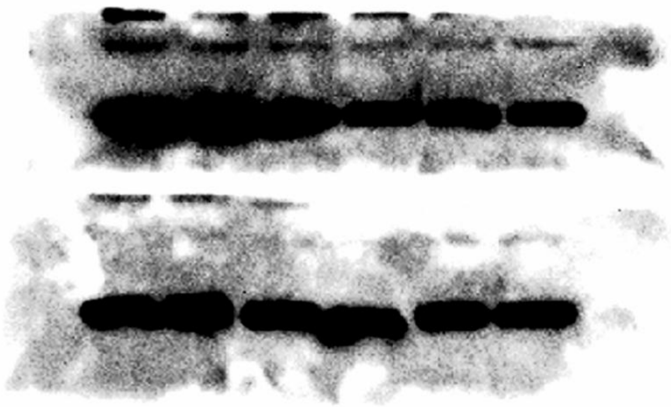

Supporting Figure 6. Original blots of Bcl-2 in Figure 3D

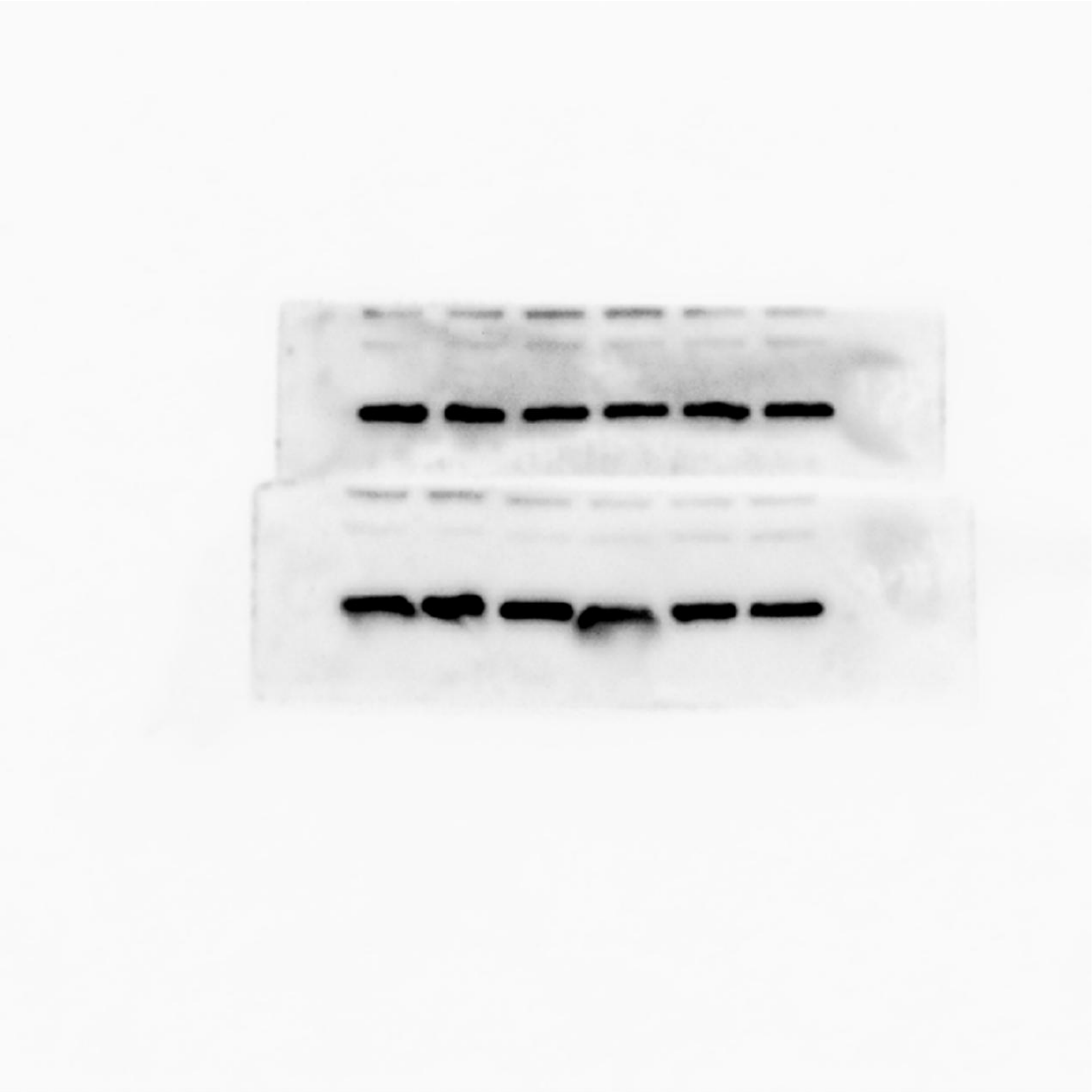

Supporting Figure 7. Original blots of Bax in Figures 3C and D

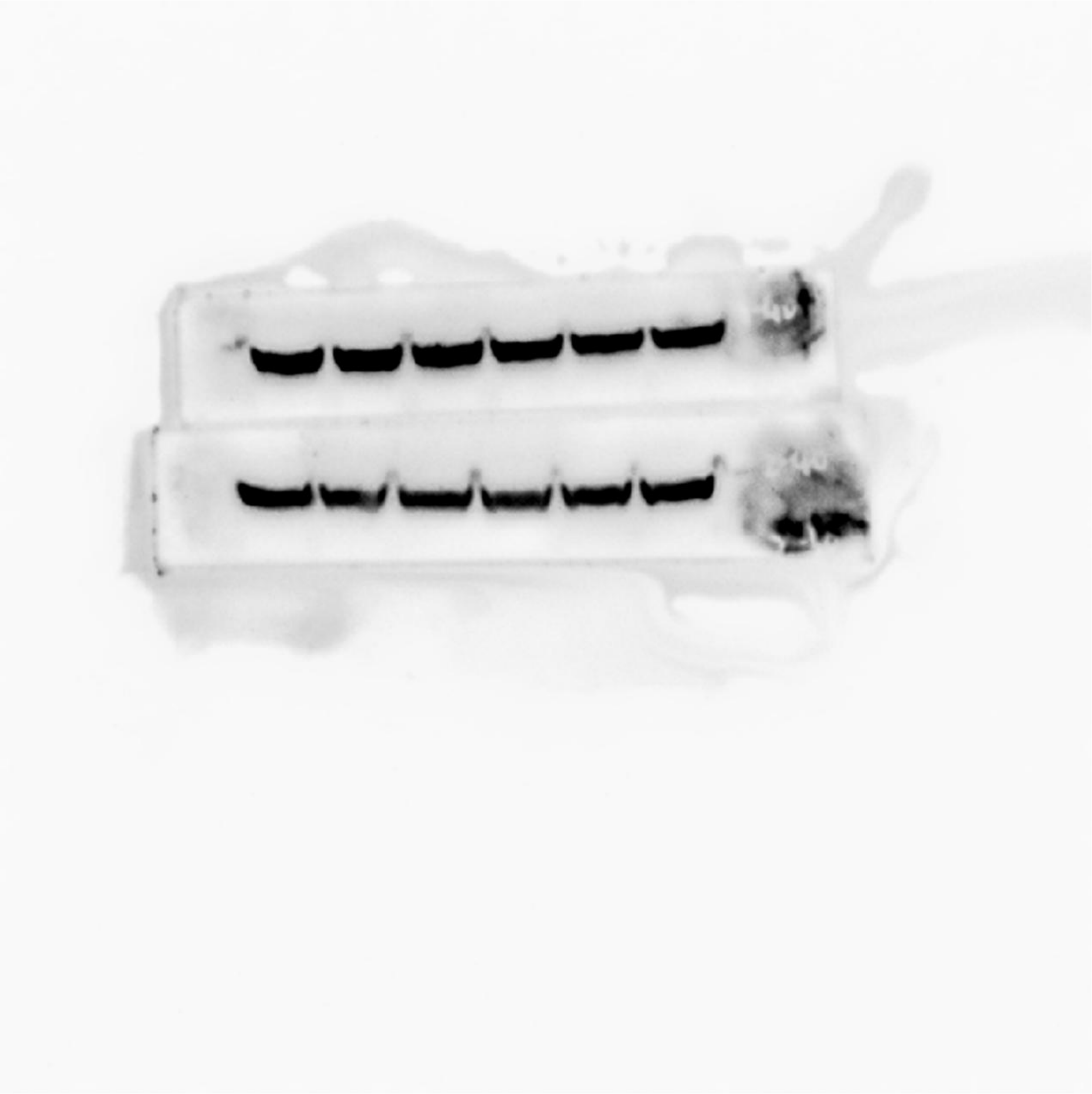

Supporting Figure 8. Original blots of GAPDH in Figures 3C and D

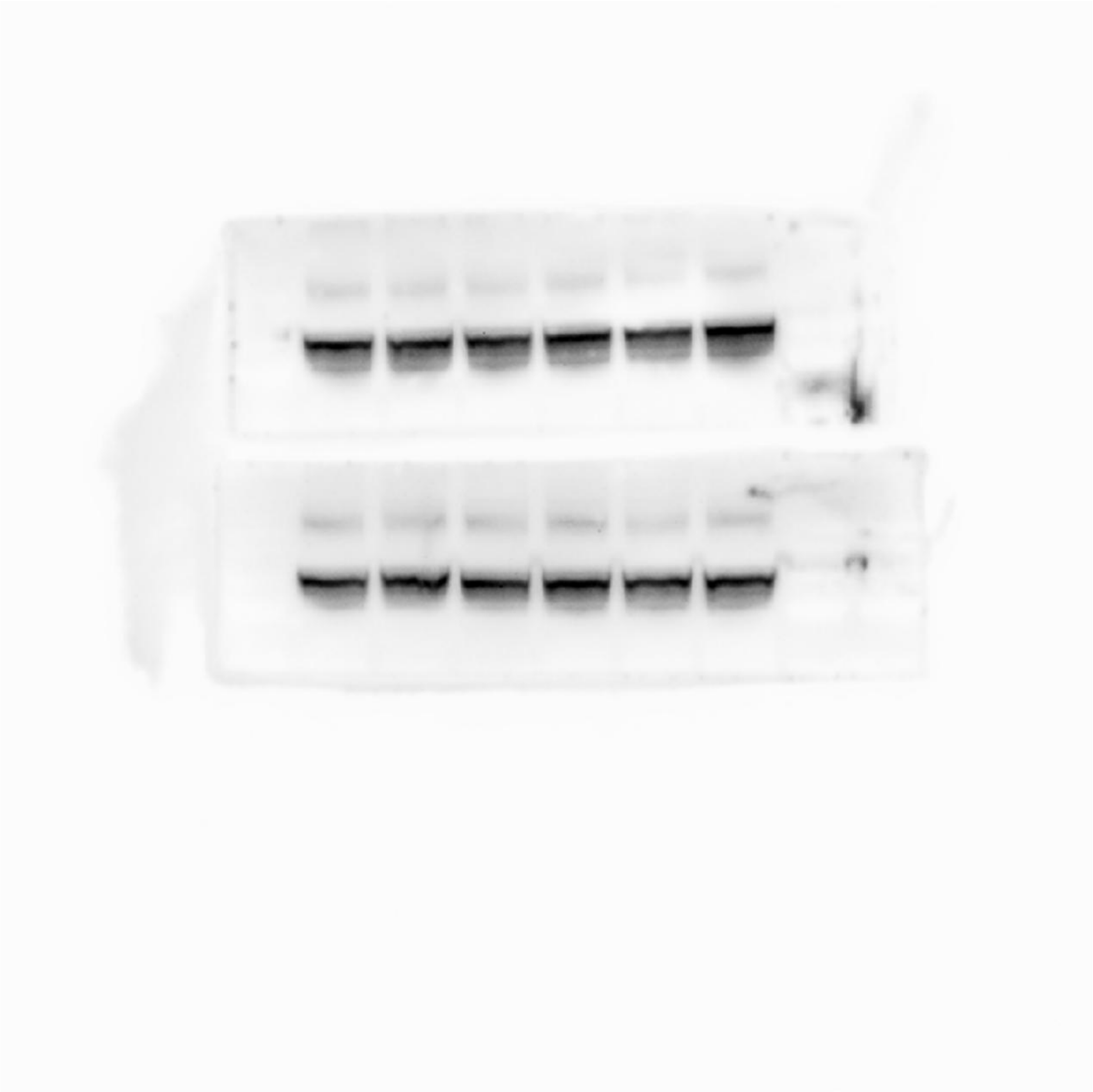

Supporting Figure 9. Original blots of β-catenin in Figures 3C and D

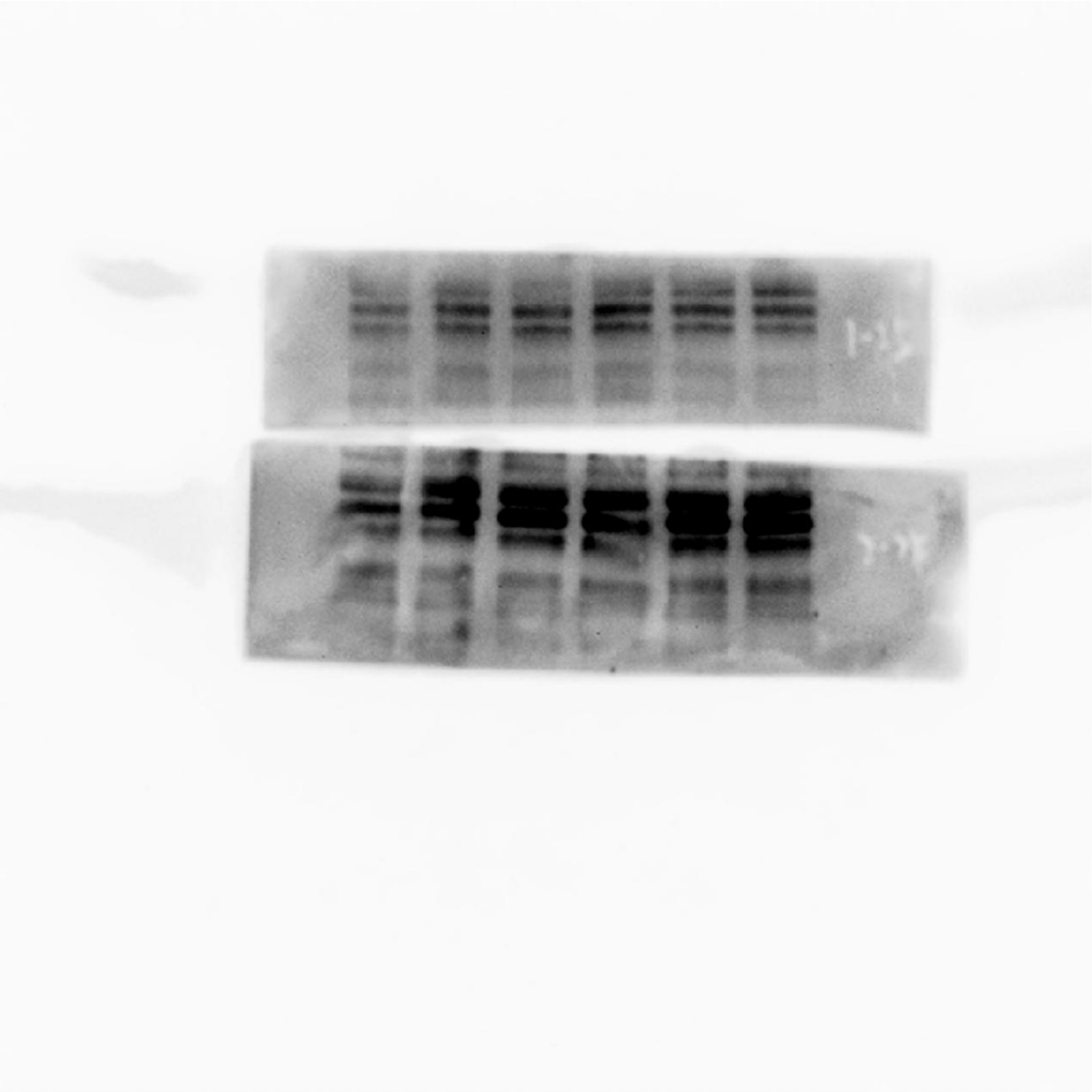

Supporting Figure 10. Original blots of p7TP3 in Figures 3D

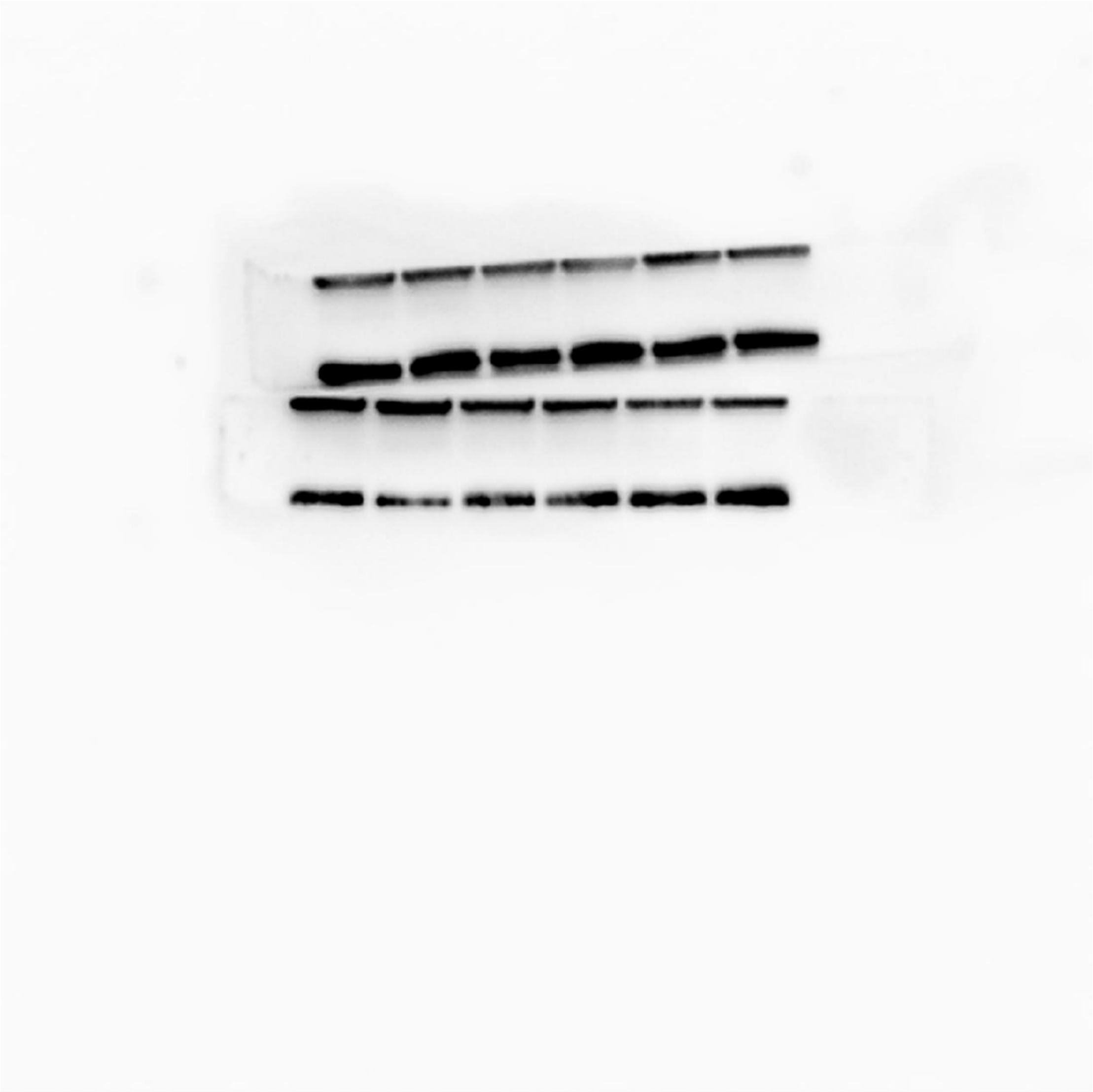

Supporting Figure 11. Original blots of Bcl-2 and Bax in Figures 3E

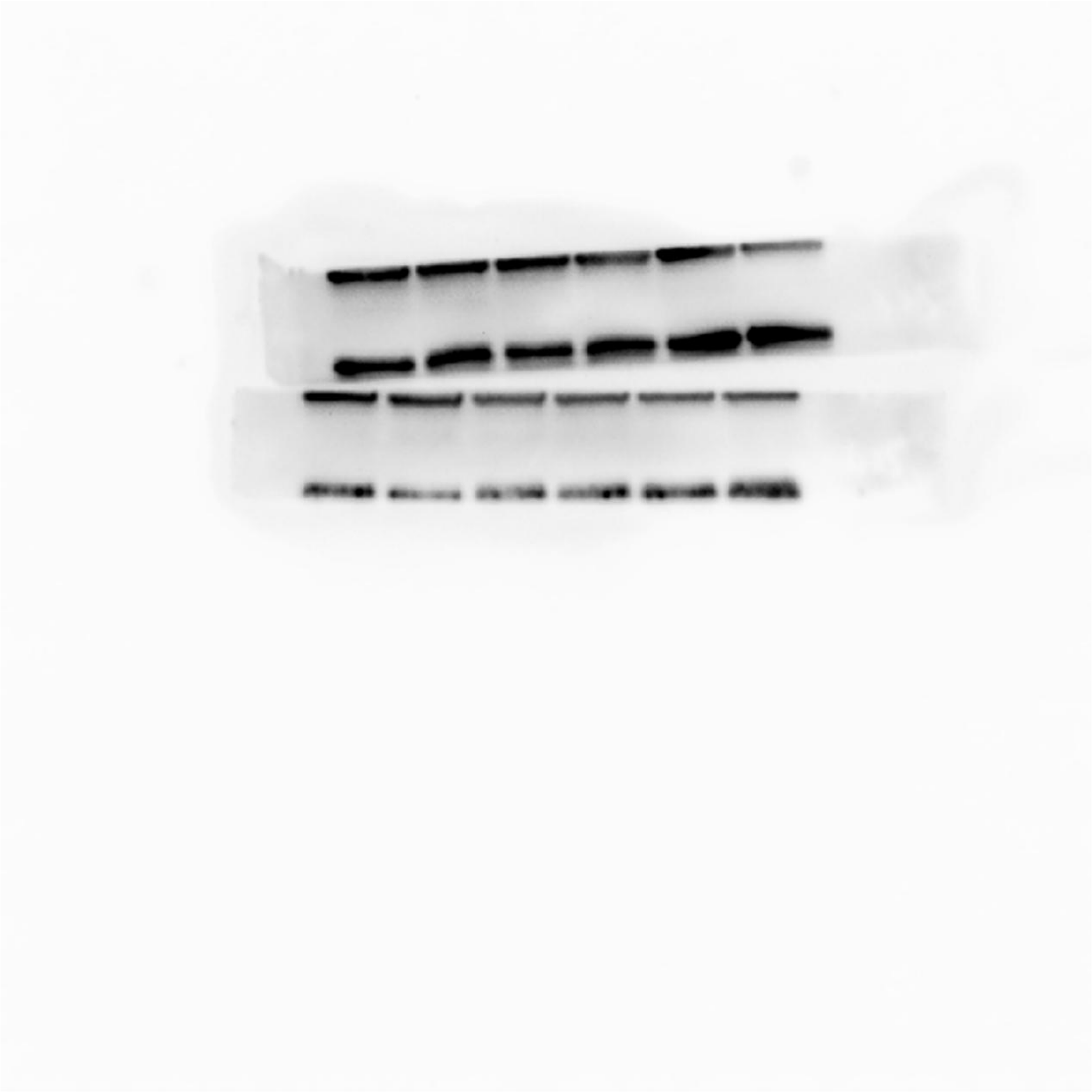

Supporting Figure 12. Original blots of Bcl-2 and Bax in Figures 3F

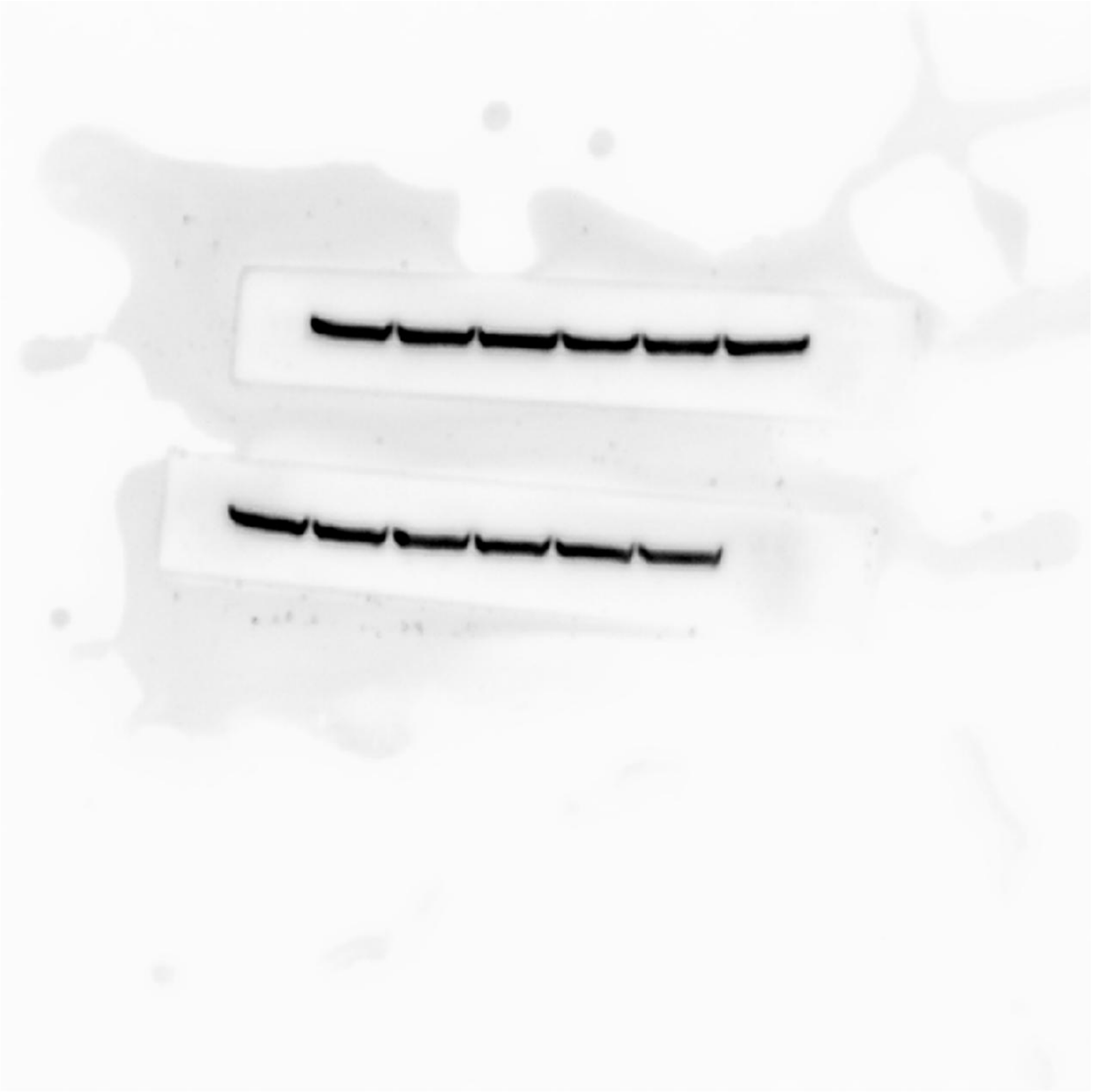

Supporting Figure 13. Original blots of GAPDF in Figures 3E and F

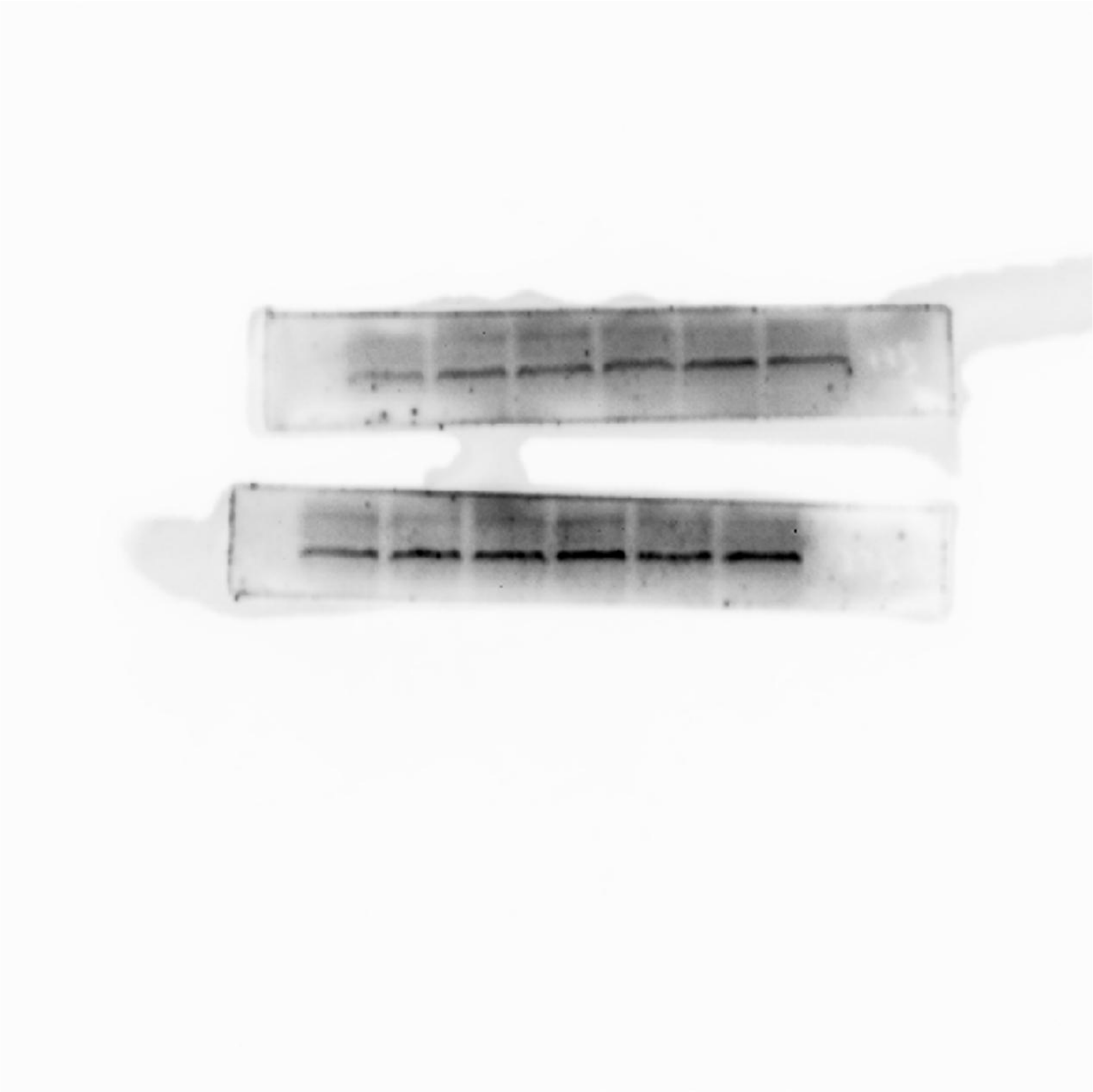

Supporting Figure 14. Original blots of p7TP3 in Figures 3E

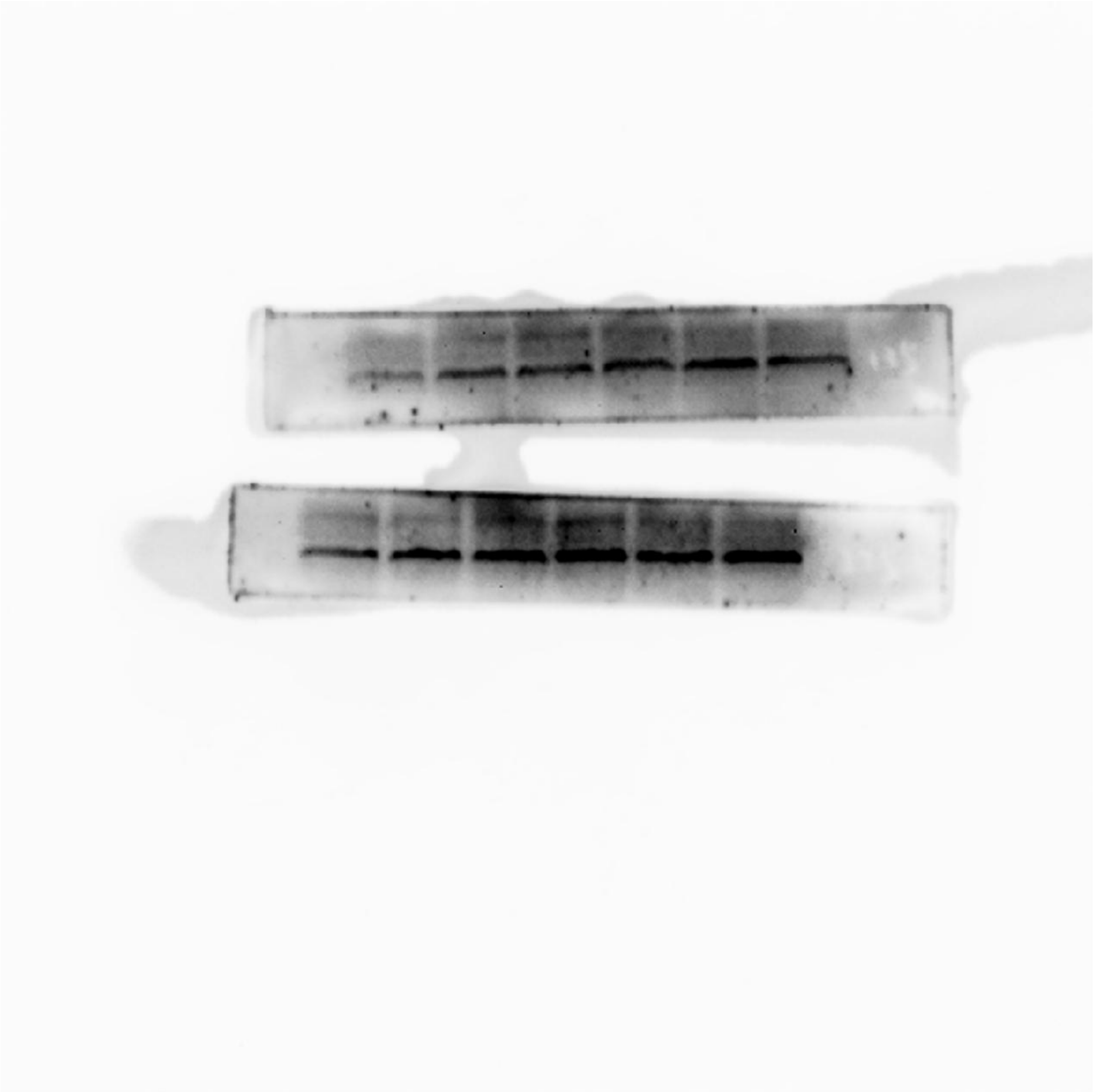

Supporting Figure 15. Original blots of p7TP3 in Figures 3F

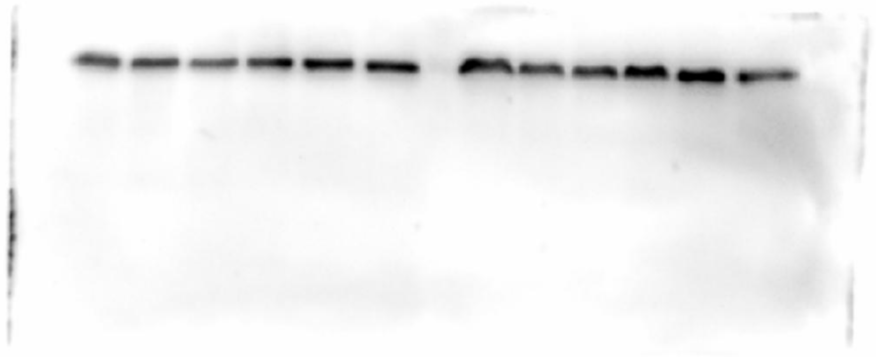

Supporting Figure 16. Original blots of p7TP3 in Figure 6A

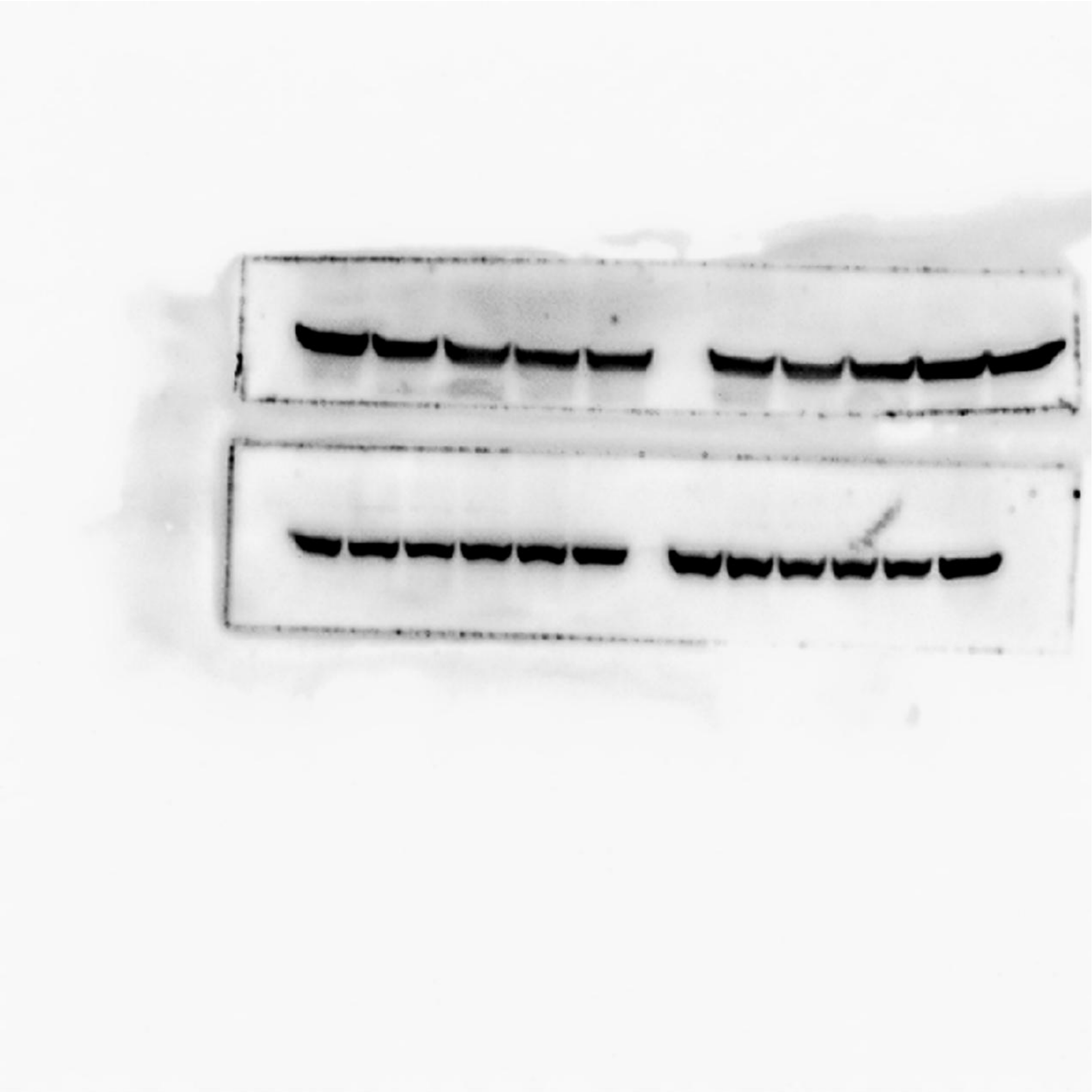

Supporting Figure 17. Original blots of GAPDH in Figure 6A

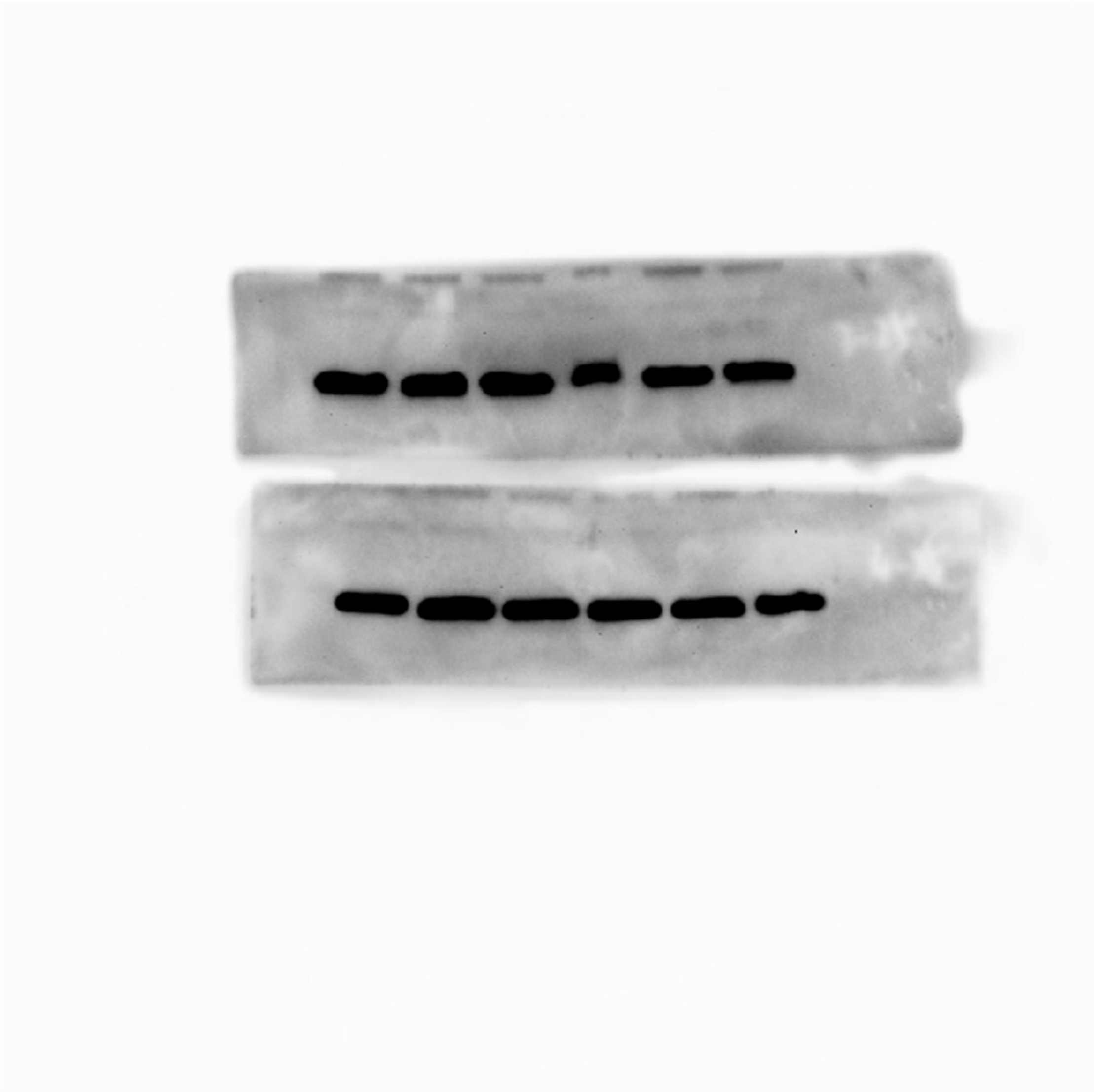

Supporting Figure 18. Original blots of HepG2-Bax in Figure S2C

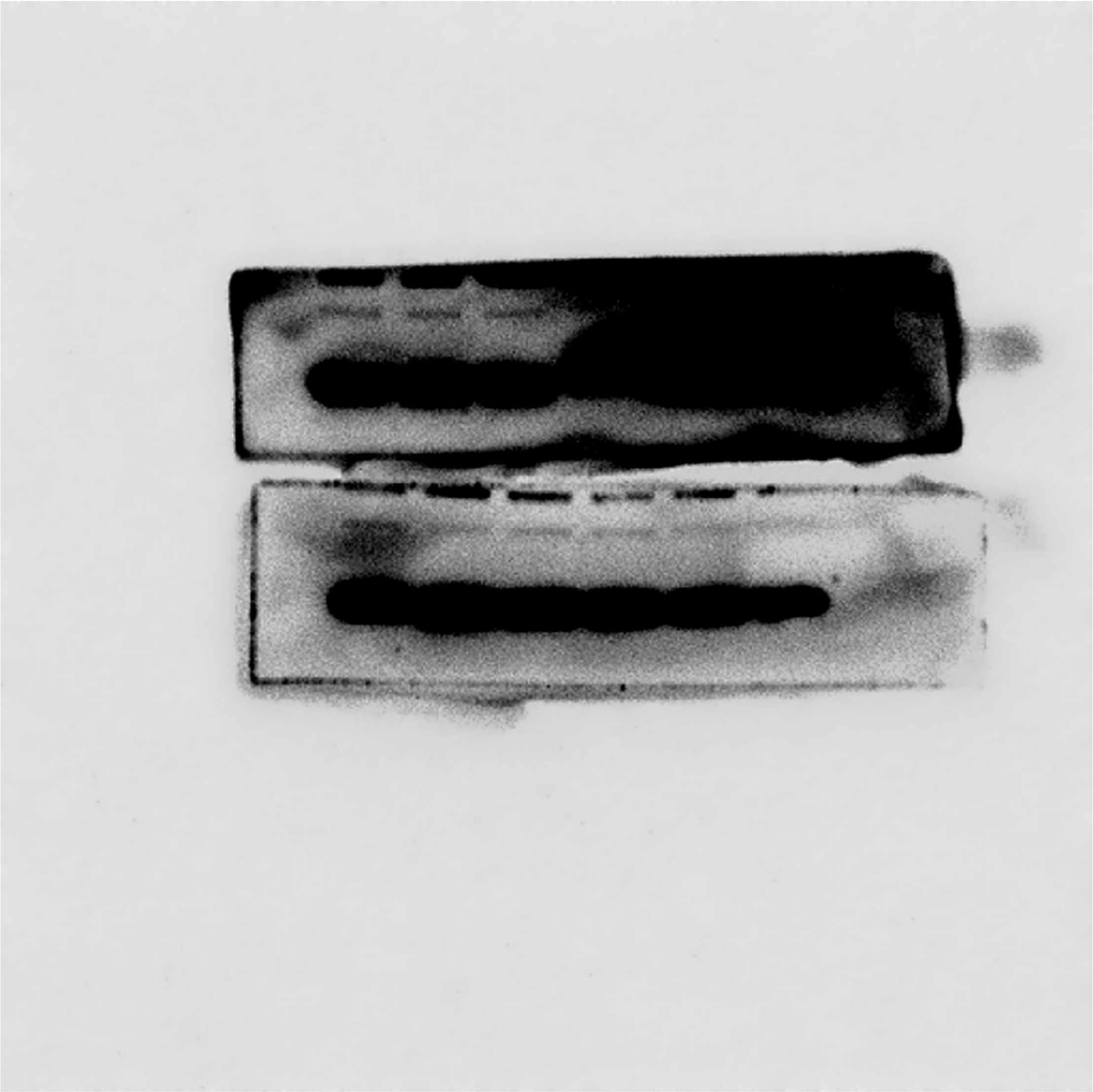

Supporting Figure 19. Original blots of HepG2-Bcl2 in Figure S2C

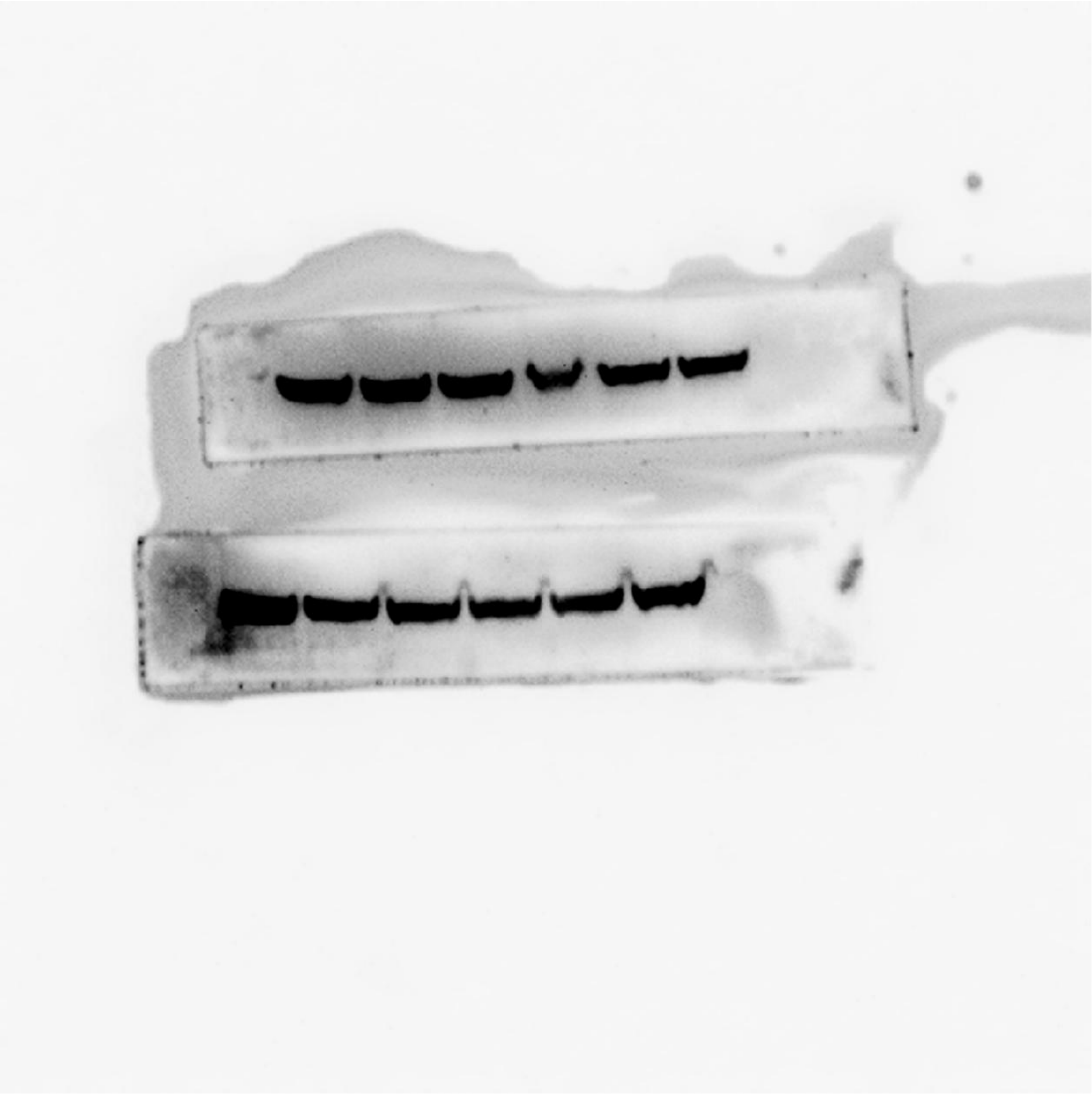

Supporting Figure 20. Original blots of HepG2-GAPDH in Figure S2C

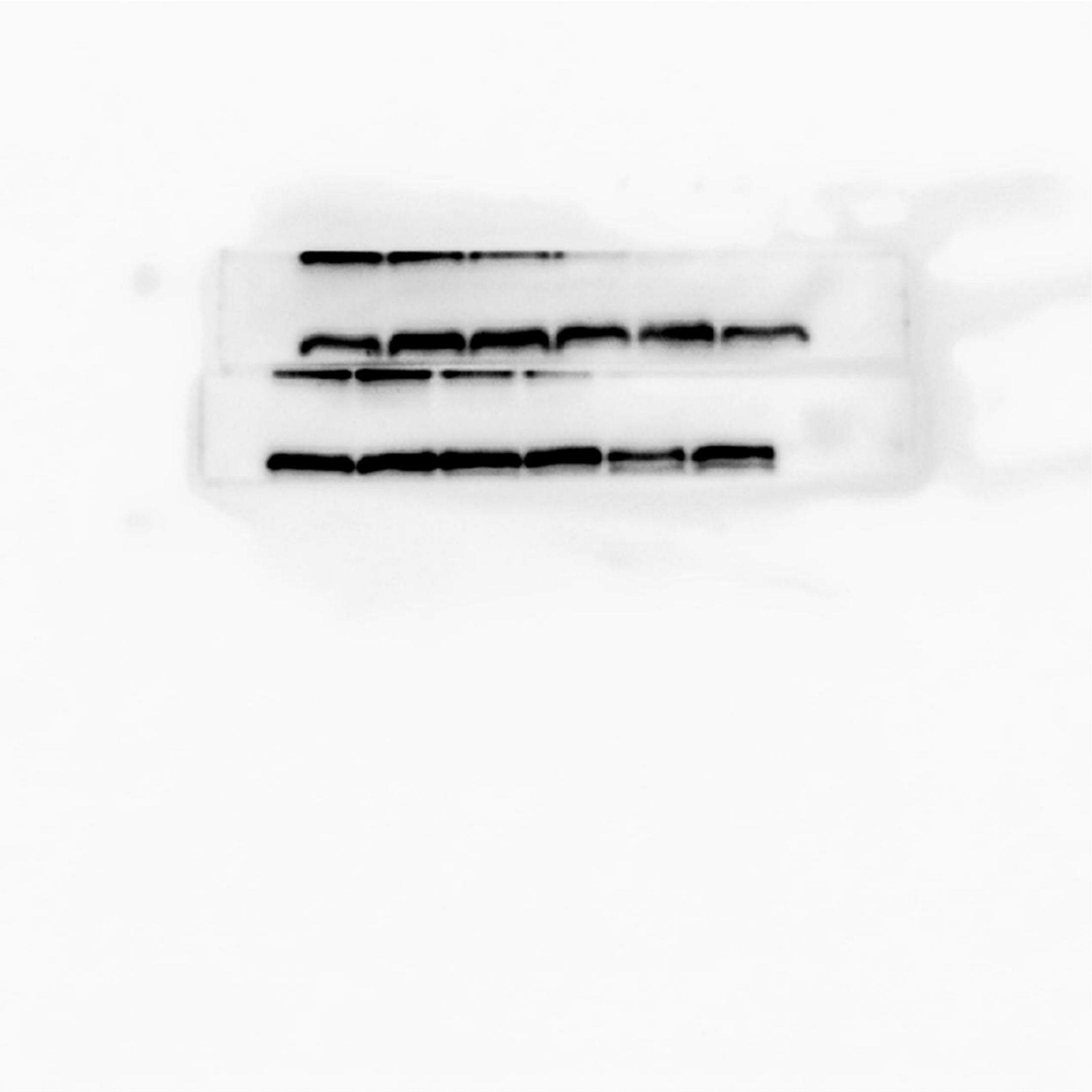

Supporting Figure 21. Original blots of Huh7-Bax in Figure S2C

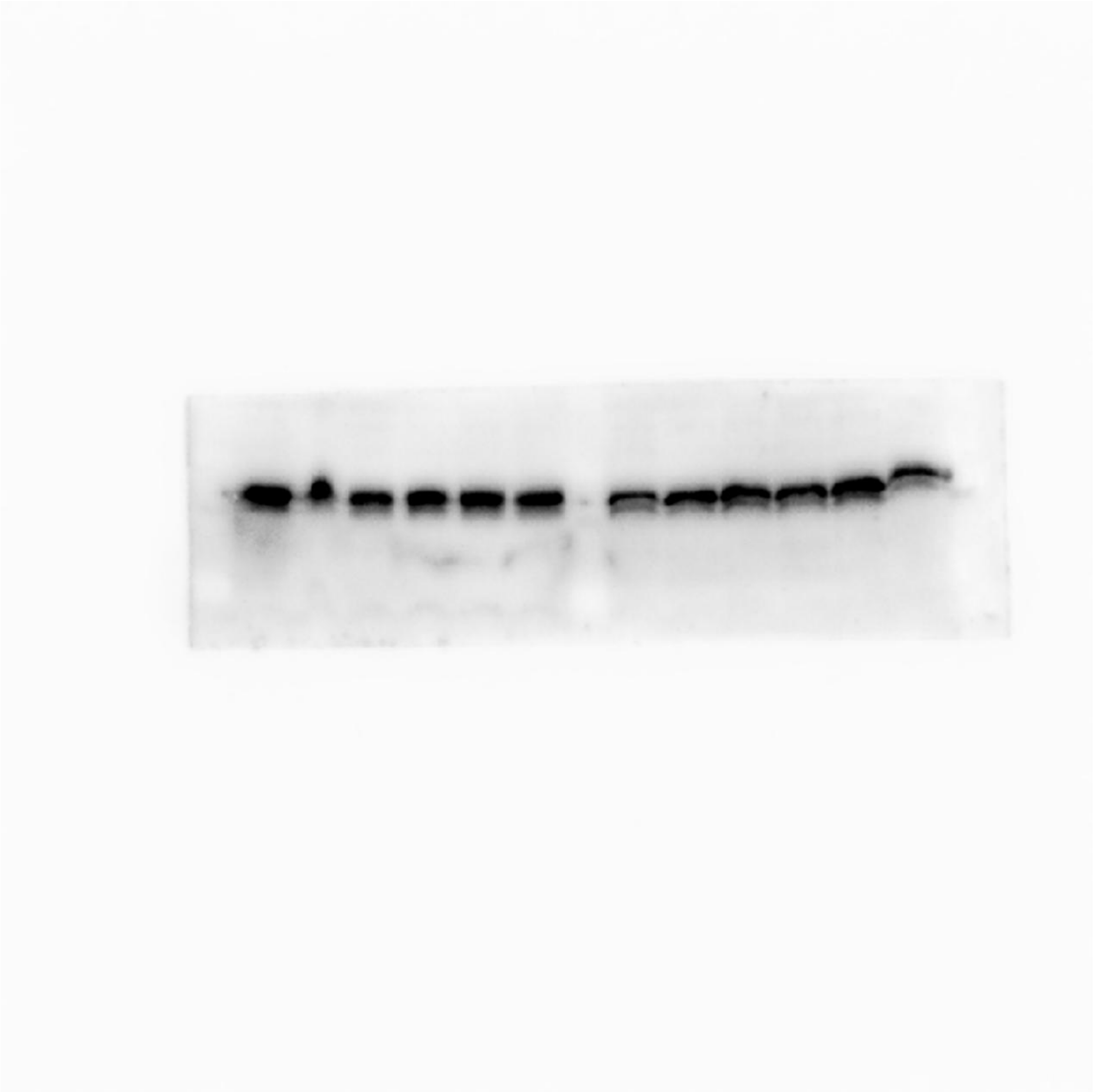

Supporting Figure 22. Original blots of Huh7-Bcl2 in Figure S2C

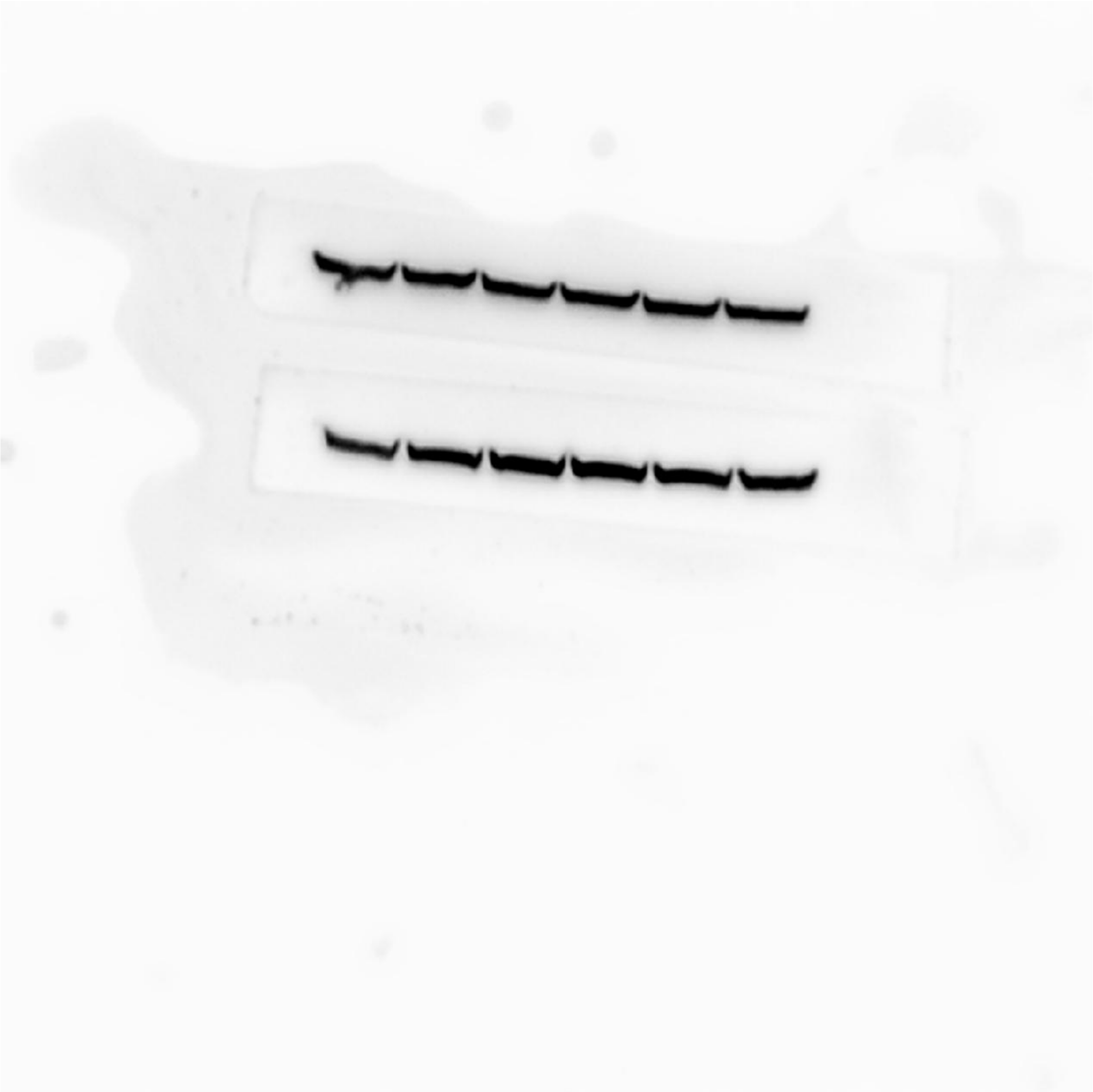

Supporting Figure 23. Original blots of Huh7-GAPDH for Bax in Figure S2C

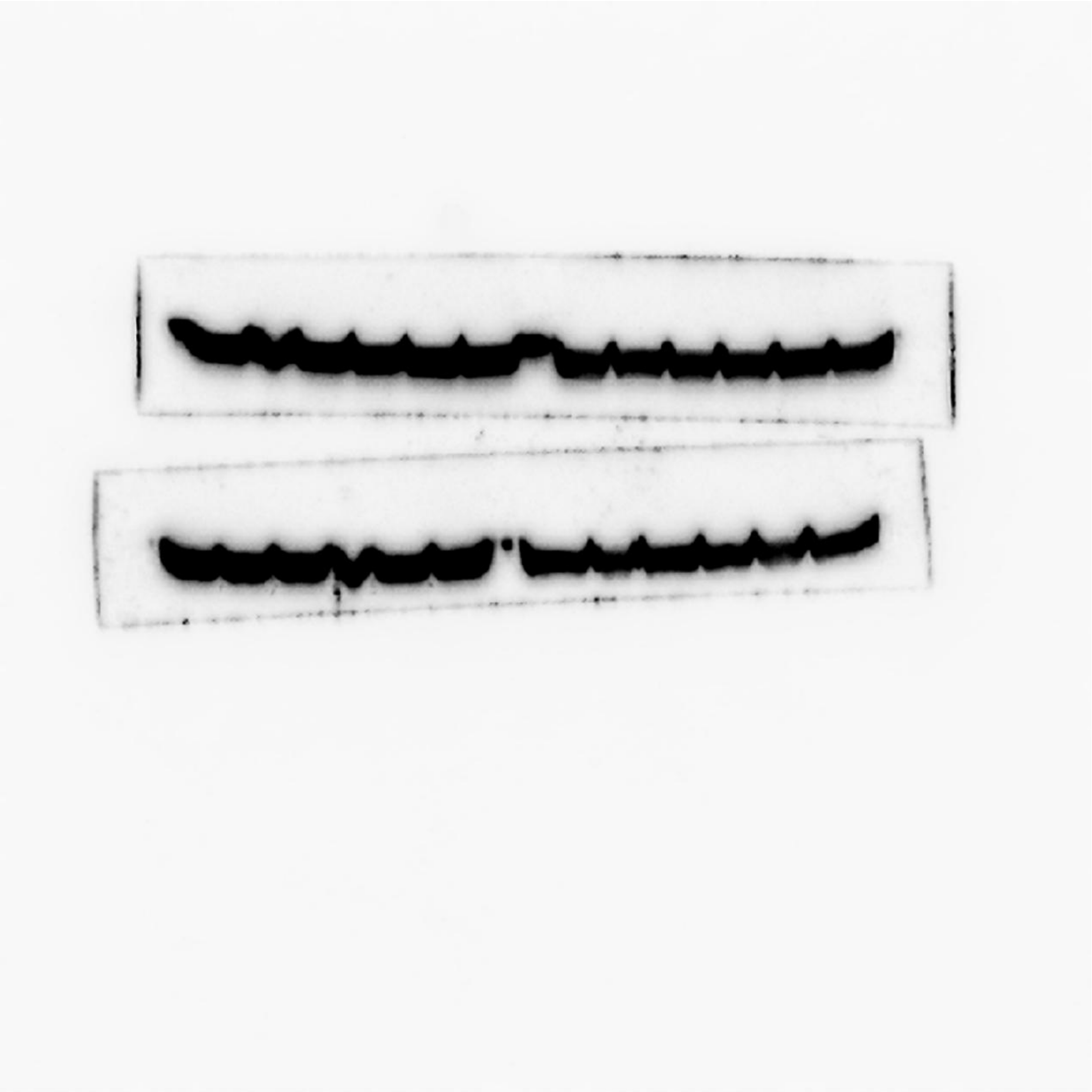

Supporting Figure 24. Original blots of Huh7-GAPDH for Bcl2 in Figure S2C

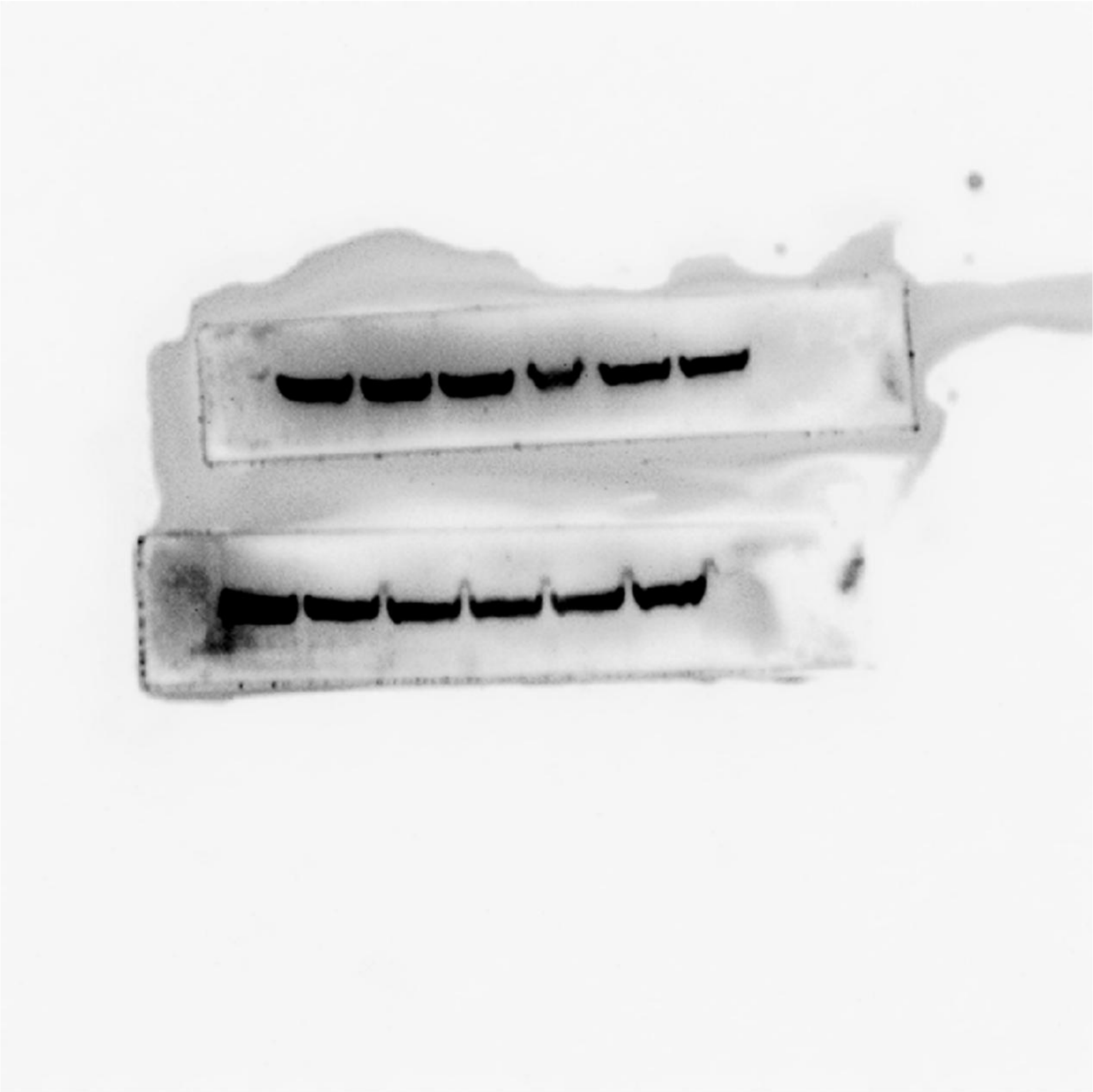

Supporting Figure 25. Original blots of HepG2-GAPDH in Figure S3A

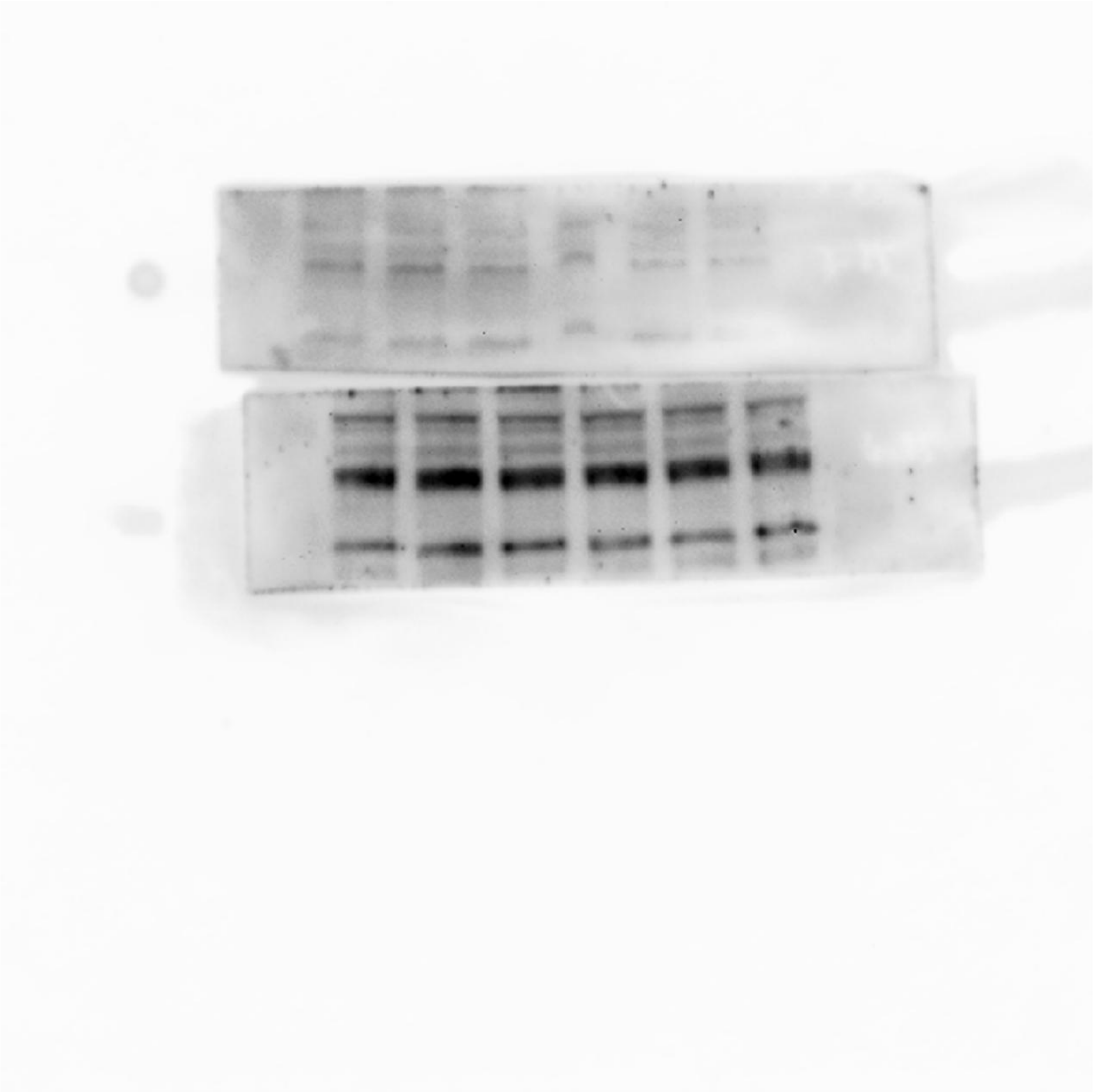

Supporting Figure 26. Original blots of HepG2-p7TP3 in Figure S3A

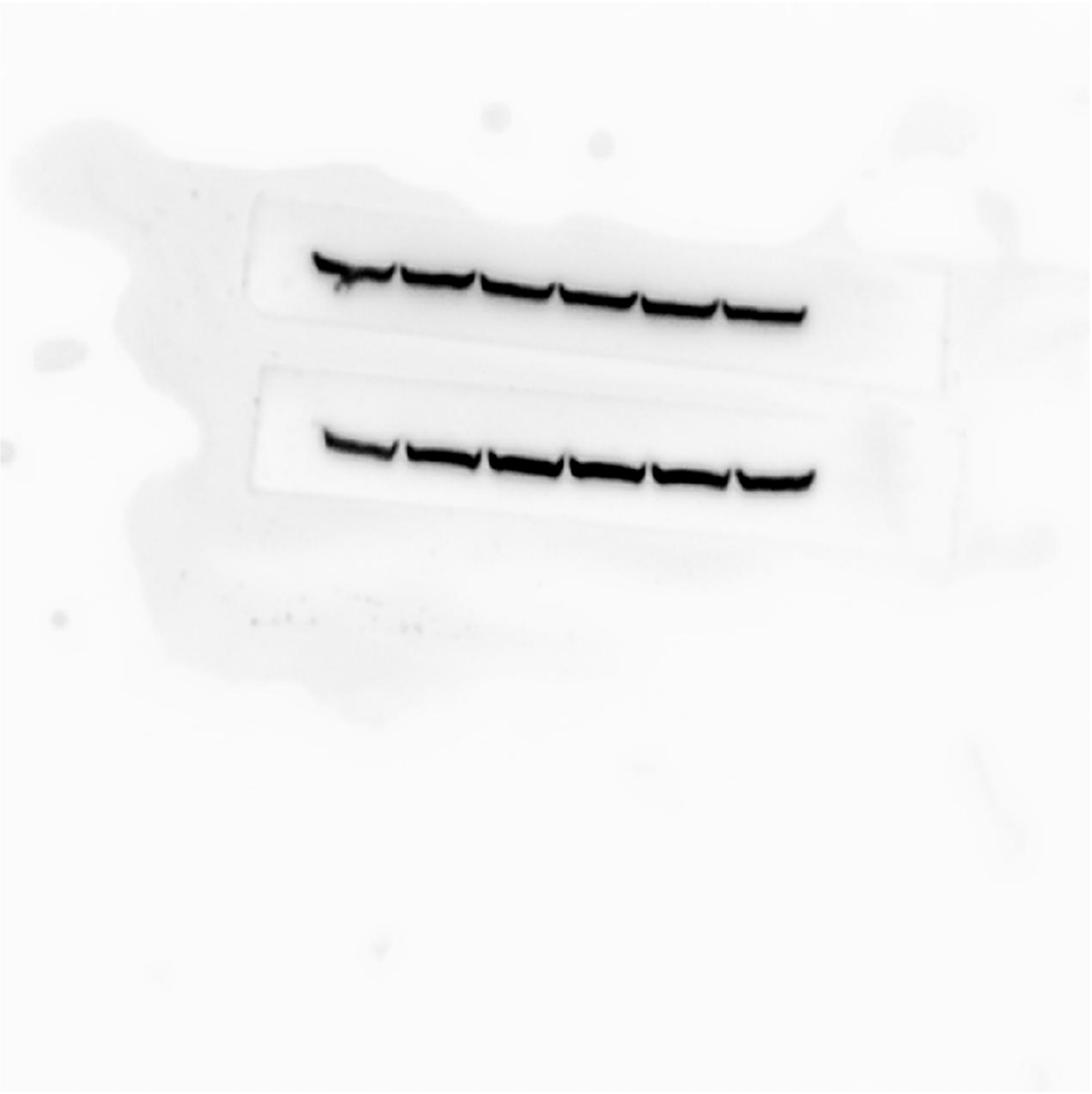

Supporting Figure 27. Original blots of Huh7-GAPDH in Figure S3B

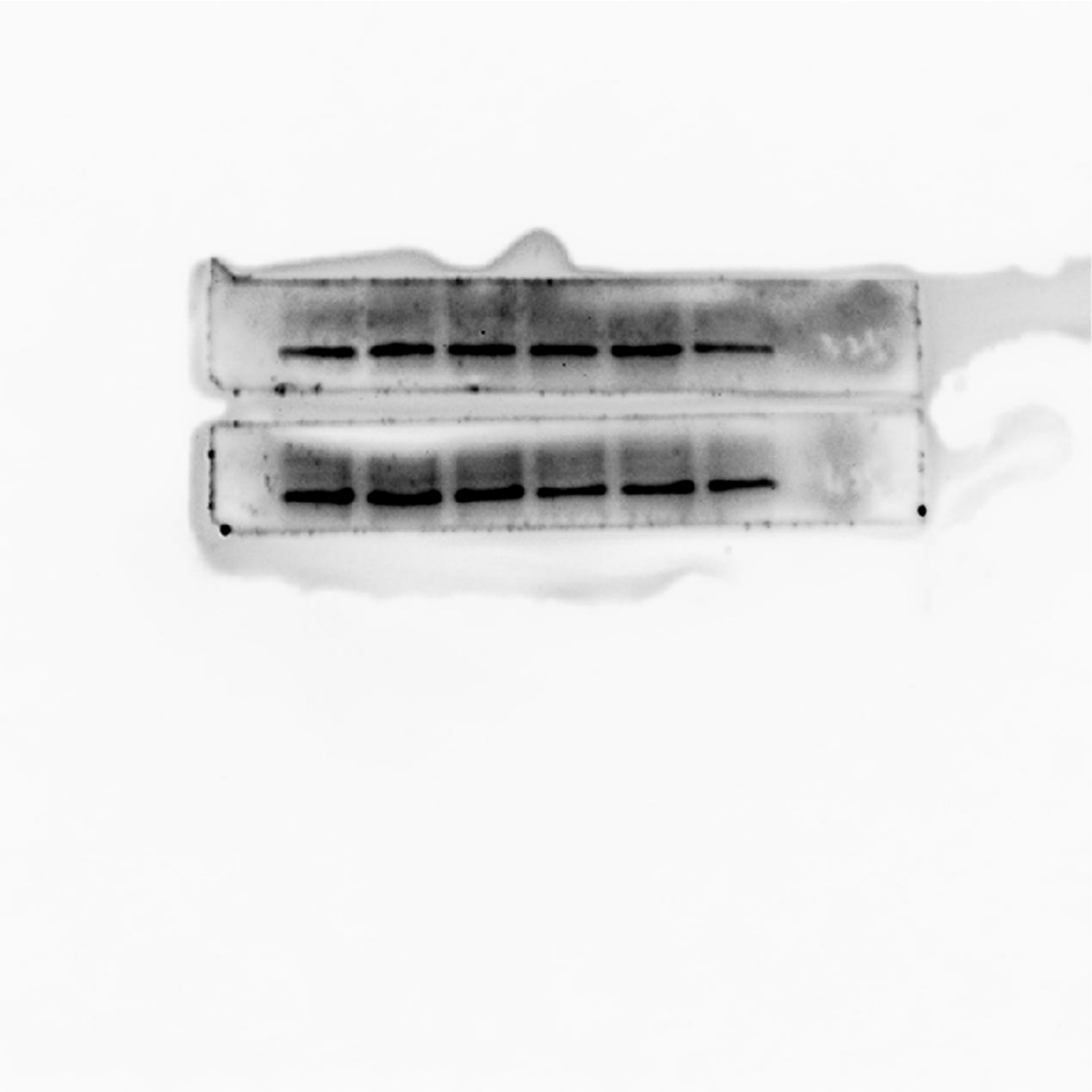

Supporting Figure 28. Original blots of Huh7-p7TP3 in Figure S3B

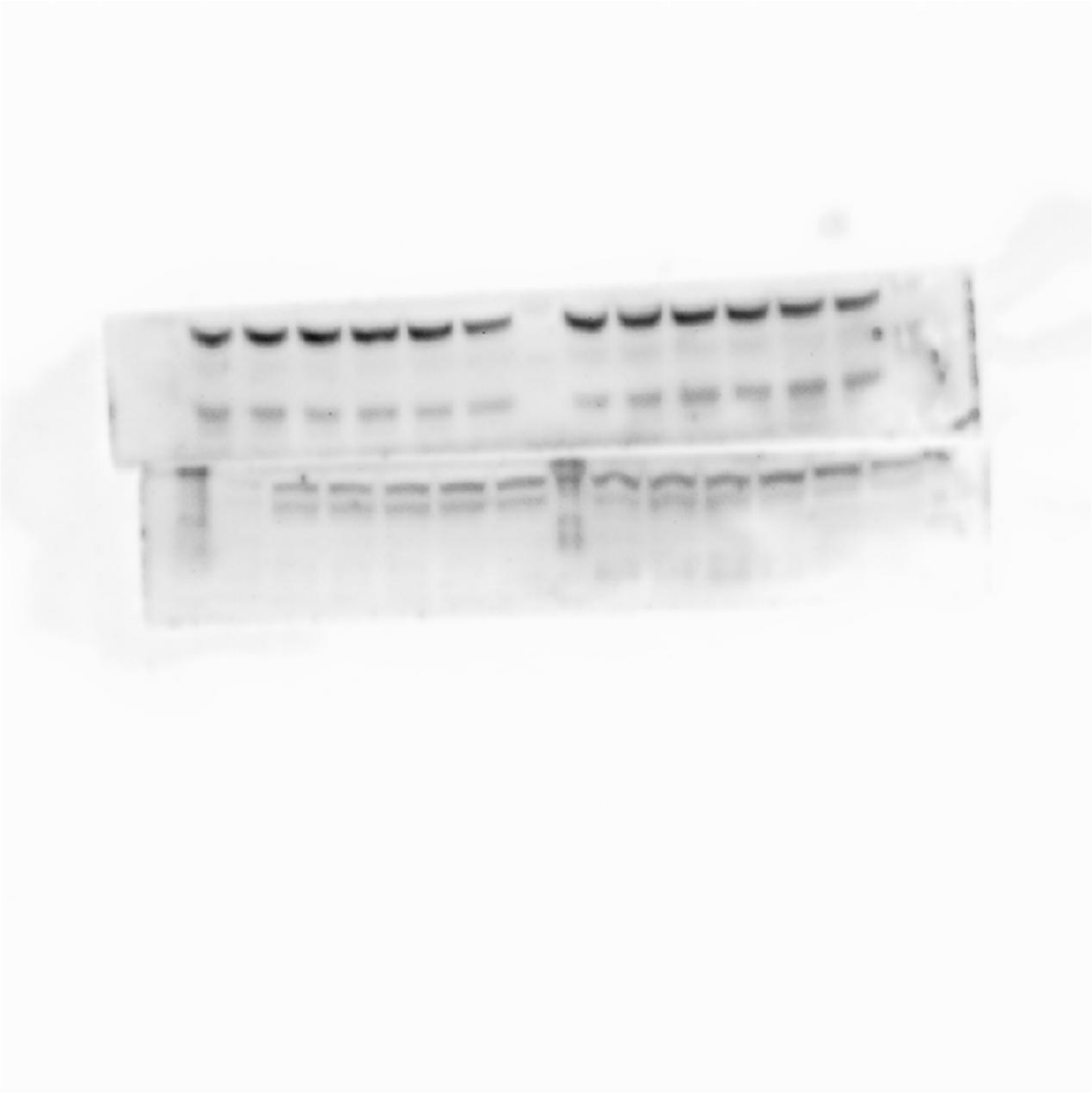

Supporting Figure 29. Original blots of caspase1 and GAPDH in Figure S4

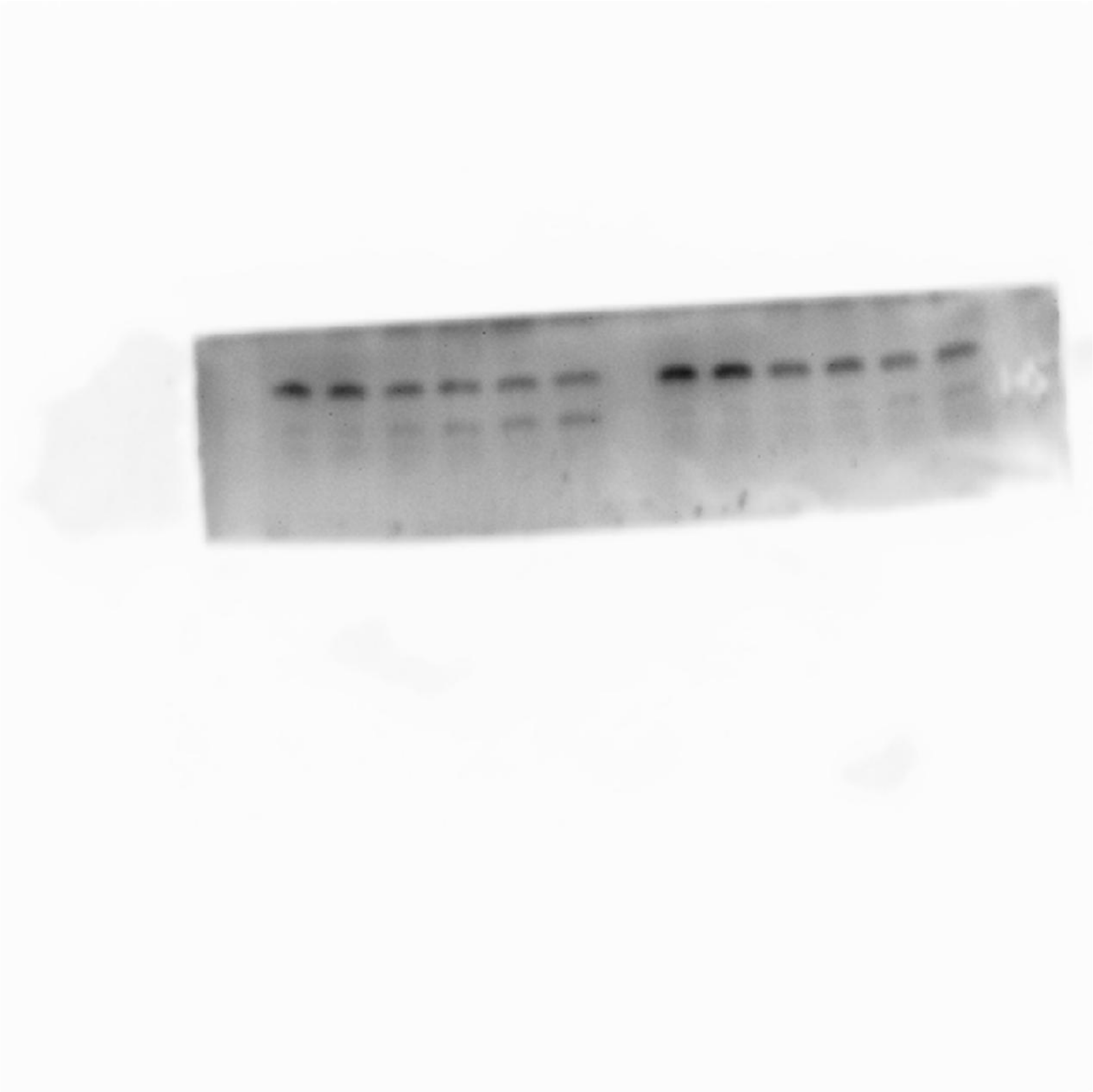

Supporting Figure 30. Original blots of IL1 in Figure S4
